# An Introduction to Topological Data Analysis: Fundamental and Practical Aspects for Data Scientists

**DOI:** 10.3389/frai.2021.667963

**Published:** 2021-09-29

**Authors:** Frédéric Chazal, Bertrand Michel

**Affiliations:** ^1^ Inria Saclay - Île-de-France Research Centre, Palaiseau, France; ^2^ Ecole Centrale de Nantes, Nantes, France

**Keywords:** topological data analysis, machine learning, geometric inference, topological inference, statistic

## Abstract

With the recent explosion in the amount, the variety, and the dimensionality of available data, identifying, extracting, and exploiting their underlying structure has become a problem of fundamental importance for data analysis and statistical learning. Topological data analysis (tda) is a recent and fast-growing field providing a set of new topological and geometric tools to infer relevant features for possibly complex data. It proposes new well-founded mathematical theories and computational tools that can be used independently or in combination with other data analysis and statistical learning techniques. This article is a brief introduction, through a few selected topics, to basic fundamental and practical aspects of tda for nonexperts.

## 1 Introduction and Motivation

Topological data analysis (tda) is a recent field that emerged from various works in applied (algebraic) topology and computational geometry during the first decade of the century. Although one can trace back geometric approaches to data analysis quite far into the past, tda really started as a field with the pioneering works of Edelsbrunner et al. (2002) and Zomorodian and Carlsson (2005) in persistent homology and was popularized in a landmark article in 2009 Carlsson (2009). tda is mainly motivated by the idea that topology and geometry provide a powerful approach to infer robust qualitative, and sometimes quantitative, information about the structure of data [e.g., Chazal (2017)].

tda aims at providing well-founded mathematical, statistical, and algorithmic methods to infer, analyze, and exploit the complex topological and geometric structures underlying data that are often represented as point clouds in Euclidean or more general metric spaces. During the last few years, a considerable effort has been made to provide robust and efficient data structures and algorithms for tda that are now implemented and available and easy to use through standard libraries such as the GUDHI library^1^ (C++ and Python) Maria et al. (2014) and its R software interface Fasy et al. (2014a), Dionysus^2^, PHAT^3^, DIPHA^4^, or Giotto^5^. Although it is still rapidly evolving, tda now provides a set of mature and efficient tools that can be used in combination with or complementarily to other data science tools.

### The Topological Data Analysis Pipeline

tda has recently known developments in various directions and application fields. There now exist a large variety of methods inspired by topological and geometric approaches. Providing a complete overview of all these existing approaches is beyond the scope of this introductory survey. However, many standard ones rely on the following basic pipeline that will serve as the backbone of this article:

1. The input is assumed to be a finite set of points coming with a notion of distance—or similarity—between them. This distance can be induced by the metric in the ambient space (e.g., the Euclidean metric when the data are embedded in $R^{d}$ ) or comes as an intrinsic metric defined by a pairwise distance matrix. The definition of the metric on the data is usually given as an input or guided by the application. It is, however, important to notice that the choice of the metric may be critical to revealing interesting topological and geometric features of the data.
2. A “continuous” shape is built on the top of the data in order to highlight the underlying topology or geometry. This is often a simplicial complex or a nested family of simplicial complexes, called a filtration, which reflects the structure of the data on different scales. Simplicial complexes can be seen as higher-dimensional generalizations of neighboring graphs that are classically built on the top of data in many standard data analysis or learning algorithms. The challenge here is to define such structures as are proven to reflect relevant information about the structure of data and that can be effectively constructed and manipulated in practice.
3. Topological or geometric information is extracted from the structures built on the top of the data. This may either result in a full reconstruction, typically a triangulation, of the shape underlying the data from which topological/geometric features can be easily extracted or in crude summaries or approximations from which the extraction of relevant information requires specific methods, such as persistent homology. Beyond the identification of interesting topological/geometric information and its visualization and interpretation, the challenge at this step is to show its relevance, in particular its stability with respect to perturbations or the presence of noise in the input data. For that purpose, understanding the statistical behavior of the inferred features is also an important question.
4. The extracted topological and geometric information provides new families of features and descriptors of the data. They can be used to better understand the data—in particular, through visualization—or they can be combined with other kinds of features for further analysis and machine learning tasks. This information can also be used to design well-suited data analysis and machine learning models. Showing the added value and the complementarity (with respect to other features) of the information provided using tda tools is an important question at this step.

### Topological Data Analysis and Statistics

Until quite recently, the theoretical aspects of TDA and topological inference mostly relied on deterministic approaches. These deterministic approaches do not take into account the random nature of data and the intrinsic variability of the topological quantity they infer. Consequently, most of the corresponding methods remain exploratory, without being able to efficiently distinguish between information and what is sometimes called the “topological noise” (see Section 6.2 further in the article).

A statistical approach to TDA means that we consider data as generated from an unknown distribution but also that the topological features inferred using TDA methods are seen as estimators of topological quantities describing an underlying object. Under this approach, the unknown object usually corresponds to the support of the data distribution (or part of it). The main goals of a statistical approach to topological data analysis can be summarized as the following list of problems:

**Topic 1:** proving consistency and studying the convergence rates of TDA methods.
**Topic 2:** providing confidence regions for topological features and discussing the significance of the estimated topological quantities.
**Topic 3:** selecting relevant scales on which the topological phenomenon should be considered, as a function of observed data.
**Topic 4:** dealing with outliers and providing robust methods for TDA.

### Applications of Topological Data Analysis in Data Science

On the application side, many recent promising and successful results have demonstrated the interest in topological and geometric approaches in an increasing number of fields such as material science (Kramar et al., 2013; Nakamura et al., 2015; Pike et al., 2020), 3D shape analysis (Skraba et al., 2010; Turner et al., 2014b), image analysis (Qaiser et al., 2019; Rieck et al., 2020), multivariate time series analysis (Khasawneh and Munch, 2016; Seversky et al., 2016; Umeda, 2017), medicine (Dindin et al., 2020), biology (Yao et al., 2009), genomics (Carrière and Rabadán, 2020), chemistry (Lee et al., 2017; Smith et al., 2021), sensor networks De Silva and Ghrist (2007), or transportation (Li et al., 2019), to name a few. It is beyond our scope to give an exhaustive list of applications of tda. On the other hand, most of the successes of tda result from its combination with other analysis or learning techniques (see Section 6.5 for a discussion and references). So, clarifying the position and complementarity of tda with respect to other approaches and tools in data science is also an important question and an active research domain.

The overall objective of this survey article is two-fold. First, it intends to provide data scientists with a brief and comprehensive introduction to the mathematical and statistical foundations of tda. For that purpose, the focus is put on a few selected, but fundamental, tools and topics, which are simplicial complexes (Section 2) and their use for exploratory topological data analysis (Section 3), geometric inference (Section 4), and persistent homology theory (Section 5), which play a central role in tda. Second, this article also aims at demonstrating how, thanks to the recent progress of software, tda tools can be easily applied in data science. In particular, we show how the Python version of the GUDHI library allows us to easily implement and use the tda tools presented in this article (Section 7). Our goal is to quickly provide the data scientist with a few basic keys—and relevant references—so that he can get a clear understanding of the basics of tda and will be able to start to use tda methods and software for his own problems and data.

Other reviews on tda can be found in the literature, which are complementary to our work. Wasserman (2018) presented a statistical view on tda, and it focused, in particular, on the connections between tda and density clustering. Sizemore et al. (2019) proposed a survey about the application of tda to neurosciences. Finally, Hensel et al. (2021) proposed a recent overview of applications of tda to machine learning.

## 2 Metric Spaces, Covers, and Simplicial Complexes

As topological and geometric features are usually associated with continuous spaces, data represented as finite sets of observations do not directly reveal any topological information *per se*. A natural way to highlight some topological structure out of data is to “connect” data points that are close to each other in order to exhibit a global continuous shape underlying the data. Quantifying the notion of closeness between data points is usually done using a distance (or a dissimilarity measure), and it often turns out to be convenient in tda to consider data sets as discrete metric spaces or as samples of metric spaces. This section introduces general concepts for geometric and topological inference; a more complete presentation of the topic is given in the study by Boissonnat et al. (2018).

### Metric Spaces

Recall that a metric space (*M*, *ρ*) is a set *M* with a function $\rho :M\times M\to R_{+}$ , called a distance, such that for any *x*, *y*, *z* ∈ *M*, the following is the case:

i) *ρ*(*x*, *y*) ≥ 0 and *ρ*(*x*, *y*) = 0 if and only if *x* = *y*,
ii) *ρ*(*x*, *y*) = *ρ*(*y*, *x*), and
iii) *ρ*(*x*, *z*) ≤ *ρ*(*x*, *y*) + *ρ*(*y*, *z*).

**FIGURE 1:**
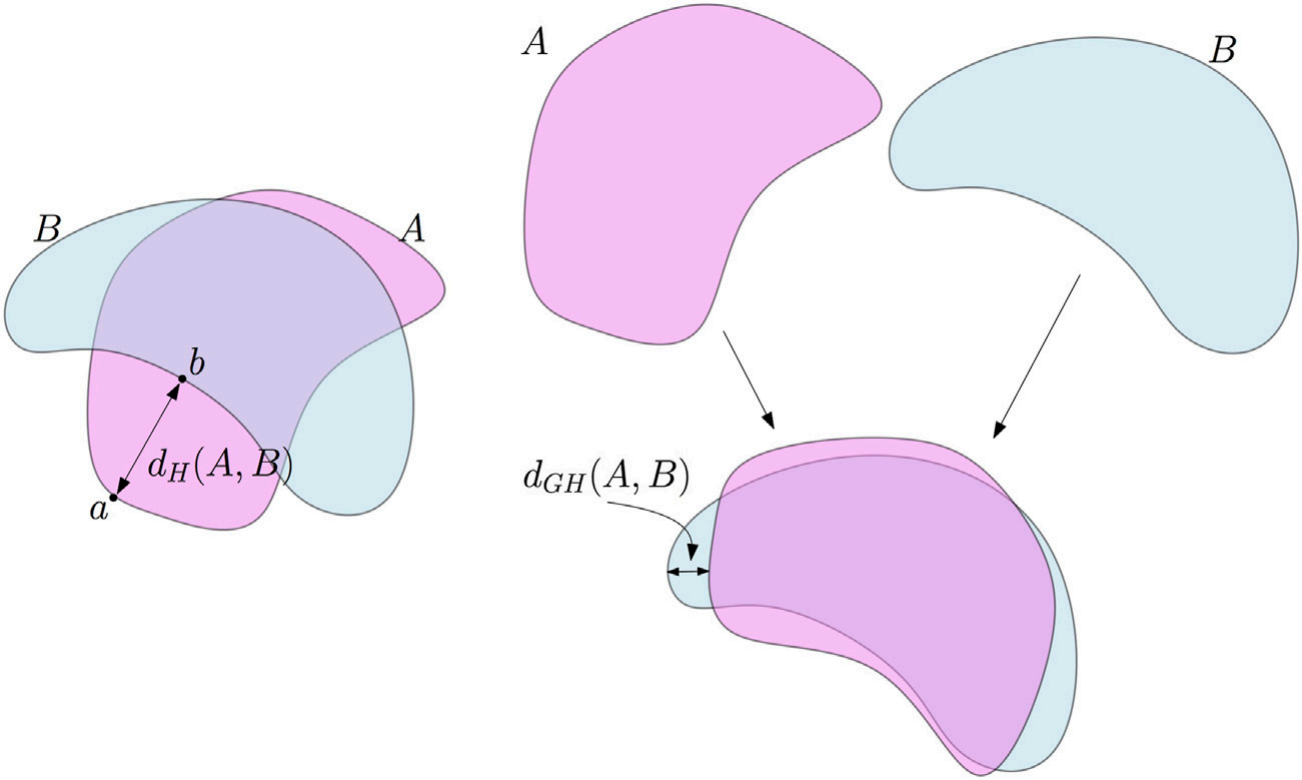
Left: the Hausdorff distance between two subsets *A* and *B* of the plane. In this example, *d*_*H*_(*A*, *B*) is the distance between the point *a* in *A* which is the farthest from *B* and its nearest neighbor *b* on *B*. Right: the Gromov–Hausdorff distance between *A* and *B*. *A* can be rotated—this is an isometric embedding of *A* in the plane—to reduce its Hausdorff distance to *B*. As a consequence, *d*_*GH*_(*A*, *B*) ≤ *d*_*H*_(*A*, *B*).

Given a metric space (*M*, *ρ*), the set K(M) of its compact subsets can be endowed with the so-called Hausdorff distance; given two compact subsets *A*, *B* ⊆ *M*, the Hausdorff distance *d*_*H*_(*A*, *B*) between *A* and *B* is defined as the smallest nonnegative number *δ* such that for any *a* ∈ *A*, there exists *b* ∈ *B* such that *ρ*(*a*, *b*) ≤ *δ*, and for any *b* ∈ *B*, there exists *a* ∈ *A* such that *ρ*(*a*, *b*) ≤ *δ* (see Figure 1). In other words, if for any compact subset *C* ⊆ *M*, we denote by $d\left(.,C\right):M\to R_{+}$ the distance function to *C* defined by *d*(*x*, *C*)≔ inf _*c*∈*C*_ *ρ*(*x*, *c*) for any *x* ∈ *M*, then one can prove that the Hausdorff distance between *A* and *B* is defined by any of the two following equalities:

$$\begin{array}{ccc} d_{H}\left(A,B\right) & = & \max\left\{\underset{b\in B}{\sup}d\left(b,A\right),\underset{a\in A}{\sup}d\left(a,B\right)\right\}\\ & = & \underset{x\in M}{\sup}|d\left(x,A\right)-d\left(x,B\right)|=\|d\left(.,A\right)-d\left(.,B\right){\|}_{\infty } \end{array}$$

It is a basic and classical result that the Hausdorff distance is indeed a distance on the set of compact subsets of a metric space. From a tda perspective, it provides a convenient way to quantify the proximity between different data sets issued from the same ambient metric space. However, it sometimes occurs that one has to compare data sets that are not sampled from the same ambient space. Fortunately, the notion of the Hausdorff distance can be generalized to the comparison of any pair of compact metric spaces, giving rise to the notion of the Gromov–Hausdorff distance.

Two compact metric spaces, (*M*_1_, *ρ*_1_) and (*M*_2_, *ρ*_2_), are isometric if there exists a bijection *ϕ*: *M*_1_ → *M*_2_ that preserves distances, that is, *ρ*_2_(*ϕ*(*x*), *ϕ*(*y*)) = *ρ*_1_(*x*, *y*) for any *x*, *y* ∈ *M*_1_. The Gromov–Hausdorff distance measures how far two metric spaces are from being isometric.

**Definition 1.** *The Gromov–Hausdorff distance d*_*GH*_(*M*_1_, *M*_2_) *between two compact metric spaces is the infimum of the real numbers r* ≥ 0 *such that there exists a metric space* (*M*, *ρ*) *and two compact subspaces C*_1_ and *C*_2_ ⊂ *M that are isometric to M*_1_ *and M*_2_ *and such that d*_*H*_(*C*_1_, *C*_2_) ≤ *r.*

The Gromov–Hausdorff distance will be used later, in Section 5, for the study of stability properties and persistence diagrams.

Connecting pairs of nearby data points by edges leads to the standard notion of the neighboring graph from which the connectivity of the data can be analyzed, for example, using some clustering algorithms. To go beyond connectivity, a central idea in TDA is to build higher-dimensional equivalents of neighboring graphs using not only connecting pairs but also (*k* + 1)-uple of nearby data points. The resulting objects, called simplicial complexes, allow us to identify new topological features such as cycles, voids, and their higher-dimensional counterpart.

### Geometric and Abstract Simplicial Complexes

Simplicial complexes can be seen as higher-dimensional generalization of graphs. They are mathematical objects that are both topological and combinatorial, a property making them particularly useful for tda.

Given a set $X=\left\{x_{0},\ldots ,x_{k}\right\}\subset R^{d}$ of *k* + 1 affinely independent points, the *k*-dimensional simplex *σ* = [*x*_0_, … , *x*_*k*_] spanned by X is the convex hull of X . The points of X are called the vertices of *σ*, and the simplices spanned by the subsets of X are called the faces of *σ*. A geometric simplicial complex *K* in $R^{d}$ is a collection of simplices such that the following are the case:

$\left.i\right)$ any face of a simplex of *K* is a simplex of *K* and

$\left.ii\right)$ the intersection of any two simplices of *K* is either empty or a common face of both.

The union of the simplices of *K* is a subset of $R^{d}$ called the underlying space of *K* that inherits from the topology of $R^{d}$ . So, *K* can also be seen as a topological space through its underlying space. Notice that once its vertices are known, *K* is fully characterized by the combinatorial description of a collection of simplices satisfying some incidence rules.

Given a set *V*, an abstract simplicial complex with the vertex set *V* is a set $\tilde{K}$ of finite subsets of *V* such that the elements of *V* belong to $\tilde{K}$ and for any $\sigma \in \tilde{K}$ , any subset of *σ* belongs to $\tilde{K}$ . The elements of $\tilde{K}$ are called the faces or the simplices of $\tilde{K}$ . The dimension of an abstract simplex is just its cardinality minus 1 and the dimension of $\tilde{K}$ is the largest dimension of its simplices. Notice that simplicial complexes of dimension 1 are graphs.

The combinatorial description of any geometric simplicial *K* obviously gives rise to an abstract simplicial complex $\tilde{K}$ . The converse is also true; one can always associate with an abstract simplicial complex $\tilde{K}$ a topological space $\left|\tilde{K}\right|$ such that if *K* is a geometric complex whose combinatorial description is the same as $\tilde{K}$ , the underlying space of *K* is homeomorphic to $\left|\tilde{K}\right|$ . Such a *K* is called a geometric realization of $\tilde{K}$ . As a consequence, abstract simplicial complexes can be seen as topological spaces and geometric complexes can be seen as geometric realizations of their underlying combinatorial structure. So, one can consider simplicial complexes at the same time as combinatorial objects that are well suited for effective computations and as topological spaces from which topological properties can be inferred.

### Building Simplicial Complexes From Data

Given a data set, or more generally, a topological or metric space, there exist many ways to build simplicial complexes. We present here a few classical examples that are widely used in practice.

**FIGURE 2:**
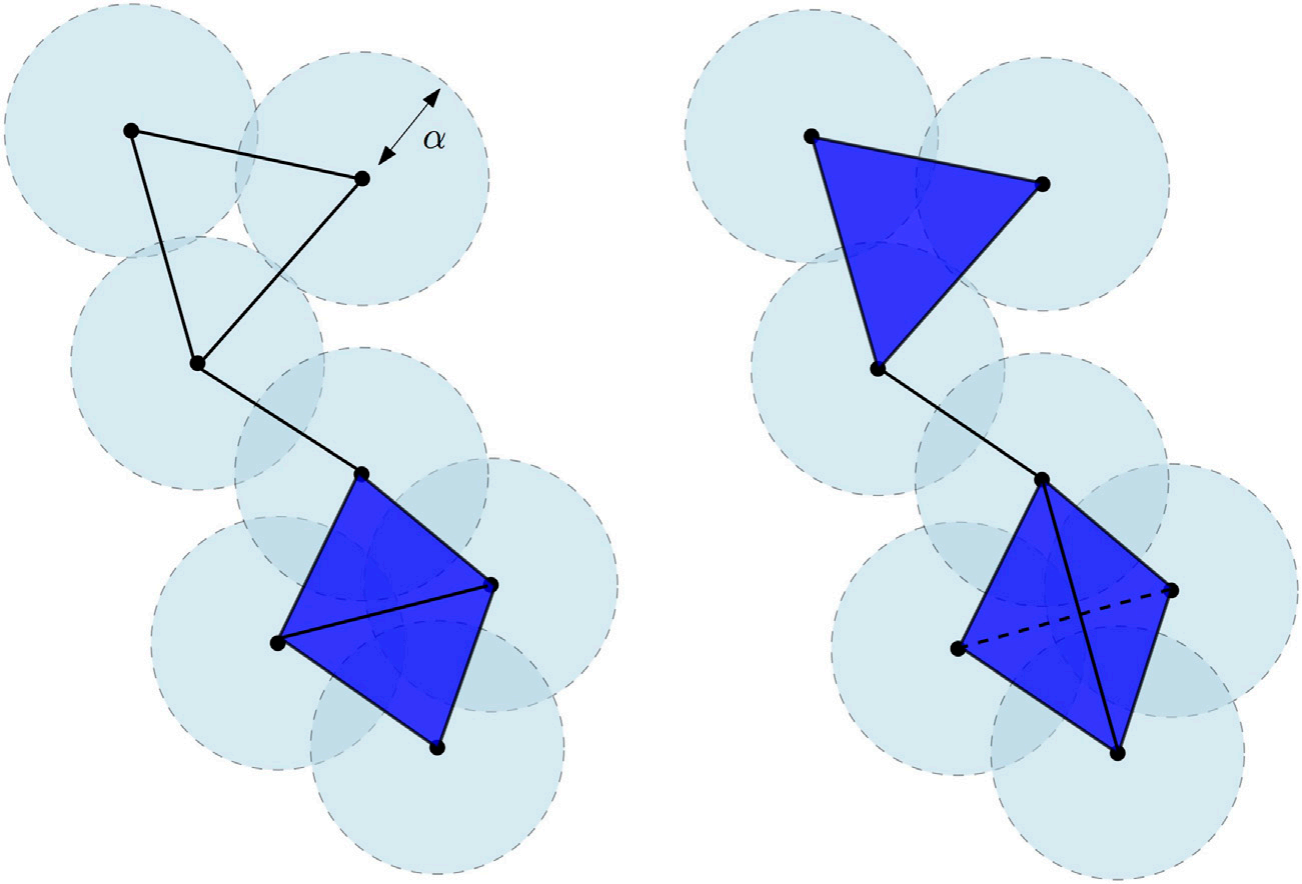
Čech complex ${\mathrm{Cech}}_{\alpha }\left(X\right)$ (left) and the Vietoris–Rips ${\mathrm{Rips}}_{2\alpha }\left(X\right)$ (right) of a finite point cloud in the plane $R^{2}$ . The bottom part of ${\mathrm{Cech}}_{\alpha }\left(X\right)$ is the union of two adjacent triangles, while the bottom part of ${\mathrm{Rips}}_{2\alpha }\left(X\right)$ is the tetrahedron spanned by the four vertices and all its faces. The dimension of the Čech complex is 2. The dimension of the Vietoris–Rips complex is 3. Notice that this latter is thus not embedded in $R^{2}$ .

A first example is an immediate extension of the notion of the *α*-neighboring graph. Assume that we are given a set of points X in a metric space (*M*, *ρ*) and a real number *α* ≥ 0. The Vietoris–Rips complex ${\mathrm{Rips}}_{\alpha }\left(X\right)$ is the set of simplices [*x*_0_, … , *x*_*k*_] such that $d_{X}\left(x_{i},x_{j}\right)\leq \alpha$ for all (*i*, *j*), see Figure 2. It follows immediately from the definition that this is an abstract simplicial complex. However, in general, even when X is a finite subset of $R^{d}$ , ${\mathrm{Rips}}_{\alpha }\left(X\right)$ does not admit a geometric realization in $R^{d}$ ; in particular, it can be of a dimension higher than *d*.

Closely related to the Vietoris–Rips complex is the Čech complex ${\mathrm{Cech}}_{\alpha }\left(X\right)$ that is defined as the set of simplices [*x*_0_, … , *x*_*k*_] such that the *k* + 1 closed balls *B*(*x*_*i*_, *α*) have a non-empty intersection, see Figure 2. Notice that these two complexes are related by

$${\mathrm{Rips}}_{\alpha }\left(X\right)\subseteq {\mathrm{Cech}}_{\alpha }\left(X\right)\subseteq {\mathrm{Rips}}_{2\alpha }\left(X\right)$$

and that if $X\subset R^{d}$ , then ${\mathrm{Cech}}_{\alpha }\left(X\right)$ and ${\mathrm{Rips}}_{2\alpha }\left(X\right)$ have the same one-dimensional skeleton, that is, the same set of vertices and edges.

### The Nerve Theorem

The Čech complex is a particular case of a family of complexes associated with covers. Given a cover $U={\left(U_{i}\right)}_{i\in I}$ of M , that is, a family of sets *U*_*i*_ such that $M=\cup_{i\in I}U_{i}$ , the nerve of U is the abstract simplicial complex C(U) whose vertices are the *U*_*i*_’s and such that

$$\sigma =\left[U_{i_{0}},\ldots ,U_{i_{k}}\right]\in C\left(U\right)\;ifandonlyif\;\overset{k}{\underset{j=0}{⋂}}U_{i_{j}}\neq \emptyset .$$

**FIGURE 3:**
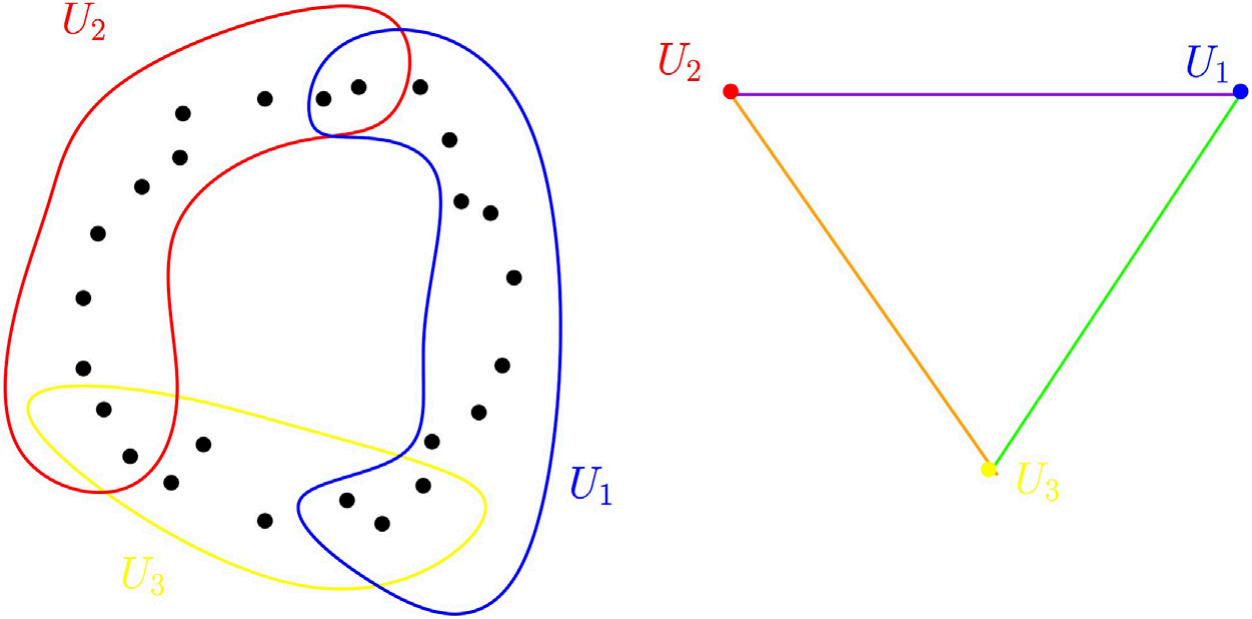
Point cloud sampled in the plane and a cover of open sets for this point cloud (left). The nerve of this cover is a triangle (right). Edges correspond to a set of the cover whereas a vertex corresponds to a non-empty intersection between two sets of the cover.

Given a cover of a data set, where each set of the cover can be, for example, a local cluster or a grouping of data points sharing some common properties, its nerve provides a compact and global combinatorial description of the relationship between these sets through their intersection patterns (see Figure 3).

A fundamental theorem in algebraic topology relates, under some assumptions, the topology of the nerve of a cover to the topology of the union of the sets of the cover. To be formally stated, this result, known as the Nerve theorem, requires the introduction of a few notions.

Two topological spaces, *X* and *Y*, are usually considered as being the same from a topological point of view if they are homeomorphic, that is, if there exist two continuous bijective maps *f*: *X* → *Y* and *g*: *Y* → *X* such that *f*°*g* and *g*°*f* are the identity map of *Y* and *X*, respectively. In many cases, asking *X* and *Y* to be homeomorphic turns out to be too strong a requirement to ensure that *X* and *Y* share the same topological features of interest for tda. Two continuous maps *f*_0_, *f*_1_: *X* → *Y* are said to be homotopic if there exists a continuous map *H*: *X* × [0, 1] → *Y* such that for any *x* ∈ *X*, *H*(*x*, 0) = *f*_0_(*x*) and *H*(*x*, 1) = *g*(*x*). The spaces *X* and *Y* are then said to be homotopy equivalent if there exist two maps, *f*: *X* → *Y* and *g*: *Y* → *X*, such that *f*°*g* and *g*°*f* are homotopic to the identity map of *Y* and *X*, respectively. The maps *f* and *g* are then called homotopy equivalent. The notion of homotopy equivalence is weaker than the notion of homeomorphism; if *X* and *Y* are homeomorphic, then they are obviously homotopy equivalent, but the converse is not true. However, spaces that are homotopy equivalent still share many topological invariants; in particular, they have the same homology (see Section 4).

A space is said to be contractible if it is homotopy equivalent to a point. Basic examples of contractible spaces are the balls and, more generally, the convex sets in $R^{d}$ . Open covers for whom all elements and their intersections are contractible have the remarkable following property.

**Theorem 1** (Nerve theorem). *Let* $U={\left(U_{i}\right)}_{i\in I}$ *be a cover of a topological space X by open sets such that the intersection of any subcollection of the U*_*i*_ *’s is either empty or contractible. Then, X and the nerve* C(U) *are homotopy equivalent.*

It is easy to verify that convex subsets of Euclidean spaces are contractible. As a consequence, if $U={\left(U_{i}\right)}_{i\in I}$ is a collection of convex subsets of $R^{d}$ , then C(U) and ∪_*i*∈*I*_ *U*_*i*_ are homotopy equivalent. In particular, if X is a set of points in $R^{d}$ , then the Čech complex ${\mathrm{Cech}}_{\alpha }\left(X\right)$ is homotopy equivalent to the union of balls $\cup_{x\in X}B\left(x,\alpha \right)$ .

The Nerve theorem plays a fundamental role in tda; it provides a way to encode the topology of continuous spaces into abstract combinatorial structures that are well suited for the design of effective data structures and algorithms.

## 3 Using Covers and Nerves for Exploratory Data Analysis and Visualization: The Mapper Algorithm

Using the nerve of covers as a way to summarize, visualize, and explore data is a natural idea that was first proposed for tda in the study by Singh et al. (2007), giving rise to the so-called Mapper algorithm.

**Definition 2.** *Let* $f:X\to R^{d}$ *, d* ≥ 1*, be a continuous real valued function and let* $U={\left(U_{i}\right)}_{i\in I}$ *be a cover of* $R^{d}$ *. The pull-back cover of X induced by* (f,U) *is the collection of open sets* ${\left(f^{-1}\left(U_{i}\right)\right)}_{i\in I}$ *. The refined pull-back is the collection of connected components of the open sets f* ^−1^(*U*_*i*_)*, i* ∈ *I.*

**FIGURE 4:**
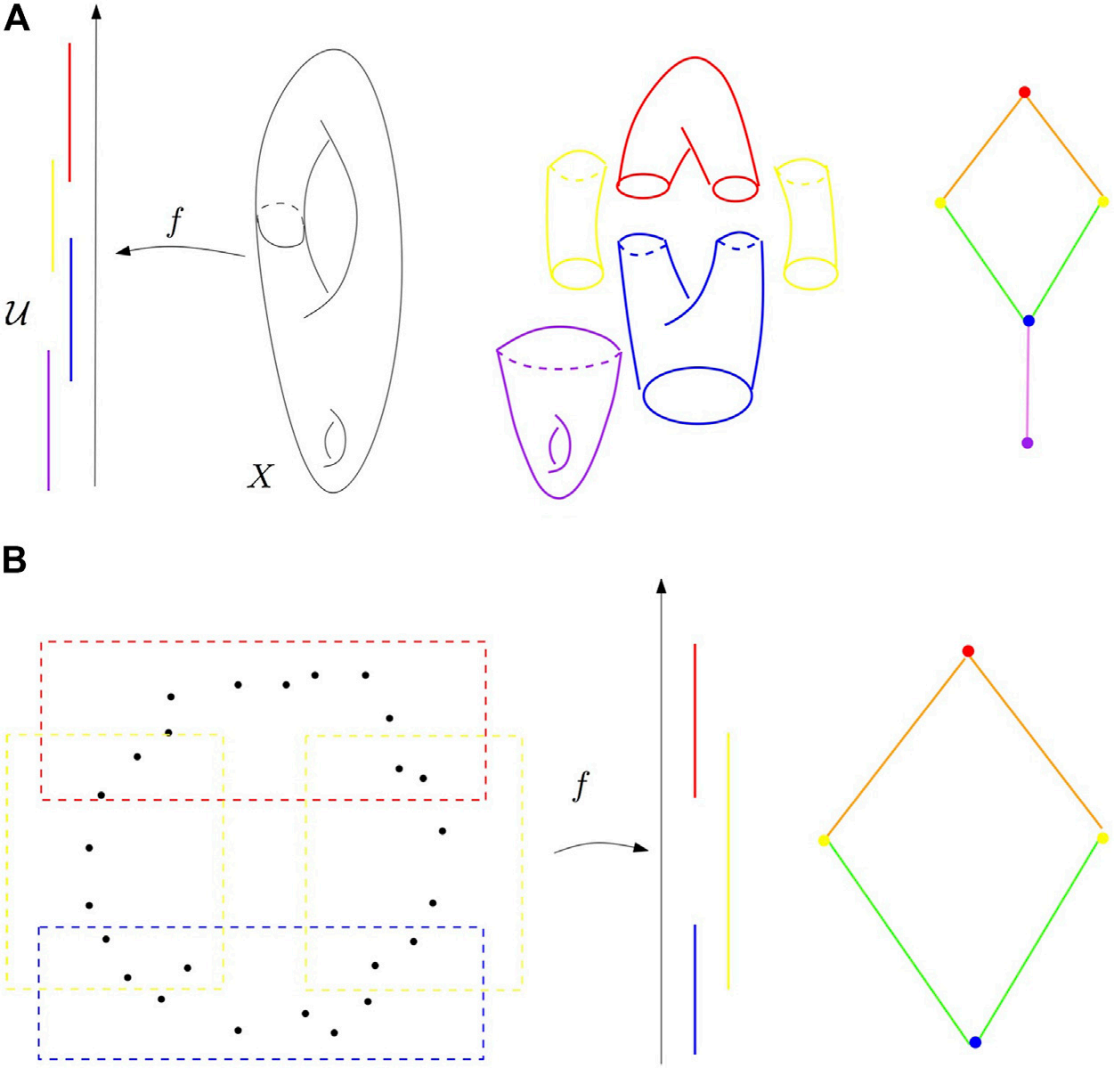
**(A)** Refined pull-back cover of the height function on a surface in $R^{3}$ and its nerve. **(B)** Mapper algorithm on a point cloud sampled around a circle and the height function. First, the pull-back cover of the height function defined on the point cloud is computed and refined (left). Second, the nerve of the refined pull-back is visualized as a graph (right).

The idea of the Mapper algorithm is, given a data set X and a well-chosen real-valued function $f:X\to R^{d}$ , to summarize X through the nerve of the refined pull-back of a cover U of f(X) (see Figure 4A). For well-chosen covers U (see below), this nerve is a graph providing an easy and convenient way to visualize the summary of the data. It is described in Algorithm 1 and illustrated on a simple example in Figure 4B.

The Mapper algorithm is very simple (see Algorithm 1); but it raises several questions about the various choices that are left to the user and that we briefly discuss in the following.

**Algorithm 1:** The Mapper algorithm

**Input:** a data set X with a metric or a dissimilarity measure between data points, a function f:X→R (or $R^{d}$ ), and a cover U of f(X)
for each U∈U decompose *f* ^−1^(*U*) into clusters $C_{U,1},\ldots ,C_{U,k_{U}}$ .
Compute the nerve of the cover of *X* defined by the $C_{U,1},\ldots ,C_{U,k_{U}}$ , U∈U .
**Output:** a simplicial complex; the nerve (often a graph for well-chosen covers → easy to visualize) includes the following:
- a vertex *v*_*U*,*i*_ for each cluster *C*_*U*,*i*_ and
- an edge between *v*_*U*,*i*_ and *v*_*U*′,*j*_ if *C*_*U*,*i*_ ∩ *C*_*U*′,*j*_ ≠ ∅.

### The Choice of *f*

The choice of the function *f*, sometimes called the filter or lens function, strongly depends on the features of the data that one expects to highlight. The following ones are among the ones more or less classically encountered in the literature:

-Density estimates: the Mapper complex may help to understand the structure and connectivity of high-density areas (clusters).
-PCA coordinates or coordinate functions obtained from a nonlinear dimensionality reduction (NLDR) technique, eigenfunctions of graph laplacians may help to reveal and understand some ambiguity in the use of nonlinear dimensionality reductions.
-The centrality function $f\left(x\right)=\sum_{y\in X}d\left(x,y\right)$ and the eccentricity function $f\left(x\right)=\max_{y\in X}d\left(x,y\right)$ sometimes appear to be good choices that do not require any specific knowledge about the data.
-For data that are sampled around one-dimensional filamentary structures, the distance function to a given point allows us to recover the underlying topology of the filamentary structures Chazal et al. (2015d).

### The Choice of the Cover U

When *f* is a real-valued function, a standard choice is to take U to be a set of regularly spaced intervals of equal length, *r* > 0, covering the set f(X) . The real *r* is sometimes called the resolution of the cover, and the percentage *g* of overlap between two consecutive intervals is called the gain of the cover. Note that if the gain *g* is chosen below 50*%*, then every point of the real line is covered by, at most, 2 open sets of U , and the output nerve is a graph. It is important to notice that the output of Mapper is very sensitive to the choice of U , and small changes in the resolution and gain parameters may result in very large changes in the output, making the method very unstable. A classical strategy consists in exploring some range of parameters and selecting the ones that turn out to provide the most informative output from the user perspective.

### The Choice of the Clusters

The Mapper algorithm requires the clustering of the preimage of the open sets U∈U . There are two strategies to compute the clusters. A first strategy consists in applying, for each U∈U , a cluster algorithm, chosen by the user, to the preimage *f* ^−1^(*U*). A second, more global, strategy consists in building a neighboring graph on the top of the data set X , for example, a k-NN graph or a *ɛ*-graph, and, for each U∈U , taking the connected components of the subgraph with the vertex set *f* ^−1^(*U*).

### Theoretical and Statistical Aspects of Mapper

Based on the results on stability and the structure of Mapper proposed in the study by Carrière and Oudot (2017), advances toward a statistically well-founded version of Mapper have been made recently in the study by Carriere et al. (2018). Unsurprisingly, the convergence of Mapper depends on both the sampling of the data and the regularity of the filter function. Moreover, subsampling strategies can be proposed to select a complex in a Rips filtration on a convenient scale, as well as the resolution and the gain for defining the Mapper graph. The case of stochastic and multivariate filters has also been studied by Carrière and Michel (2019). An alternative description of the probabilistic convergence of Mapper, in terms of categorification, has also been proposed in the study by Brown et al. (2020). Other approaches have been proposed to study and deal with the instabilities of the Mapper algorithm in the works of Dey et al. (2016), Dey et al. (2017).

### Data Analysis With Mapper

As an exploratory data analysis tool, Mapper has been successfully used for clustering and feature selection. The idea is to identify specific structures in the Mapper graph (or complex), in particular, loops and flares. These structures are then used to identify interesting clusters or to select features or variables that best discriminate the data in these structures. Applications on real data, illustrating these techniques, may be found, for example, in the studies by Carrière and Rabadán (2020), Lum et al. (2013), Yao et al. (2009).

## 4 Geometric Reconstruction and Homology Inference

Another way to build covers and use their nerves to exhibit the topological structure of data is to consider the union of balls centered on the data points. In this section, we assume that $X_{n}=\left\{x_{0},\ldots ,x_{n}\right\}$ is a subset of $R^{d}$ , sampled i. i. d. according to a probability measure *μ* with compact support $M\subset R^{d}$ . The general strategy to infer topological information about *M* from *μ* proceeds in two steps that are discussed in the following part of this section:

1. $X_{n}$ is covered by a union of balls of a fixed radius centered on the *x*_*i*_’s. Under some regularity assumptions on *M*, one can relate the topology of this union of balls to the one of *M* and
2. from a practical and algorithmic perspective, topological features of *M* are inferred from the nerve of the union of balls, using the Nerve theorem.

In this framework, it is indeed possible to compare spaces through isotopy equivalence, a stronger notion than homeomorphism; $X\subseteq R^{d}$ and $Y\subseteq R^{d}$ are said to be (ambient) isotopic if there exists a continuous family of homeomorphisms $H:\left[0,1\right]\times R^{d}\to R^{d}$ , *H* continuous, such that for any *t* ∈ [0, 1], $H_{t}=H\left(t,.\right):R^{d}\to R^{d}$ is a homeomorphism, *H*_0_ is the identity map in $R^{d}$ , and *H*_1_(*X*) = *Y*. Obviously, if *X* and *Y* are isotopic, then they are homeomorphic. The converse is not true; a knotted circle and an unknotted circle in $R^{3}$ are not homeomorphic (notice that although this claim seems rather intuitive, its formal proof requires the use of some nonobvious algebraic topology tools).

### 4.1 Distance-Like Functions and Reconstruction

Given a compact subset *K* of $R^{d}$ and a nonnegative real number *r*, the union of balls of radius *r* centered on *K*, *K* ^*r*^ = ∪_*x*∈*K*_ *B*(*x*, *r*), called the *r*-offset of *K*, is the *r*-sublevel set of the distance function $d_{K}:R^{d}\to R$ defined by *d*_*K*_(*x*) = inf _*y*∈*K*_‖*x* − *y*‖; in other words, $K^{r}=d_{k}^{-1}\left(\left[0,r\right]\right)$ . This remark allows us to use differential properties of distance functions and to compare the topology of the offsets of compact sets that are close to each other with respect to the Hausdorff distance.

**Definition 3** (Hausdorff distance in $R^{d}$ ). *The Hausdorff distance between two compact subsets K*, *K*′ *of* $R^{d}$ *is defined by*

$$d_{H}\left(K,K^{\prime }\right)=\|d_{K}-d_{K^{\prime }}{\|}_{\infty }=\underset{x\in R^{d}}{\sup}|d_{K}\left(x\right)-d_{K^{\prime }}\left(x\right)|.$$

**FIGURE 5:**
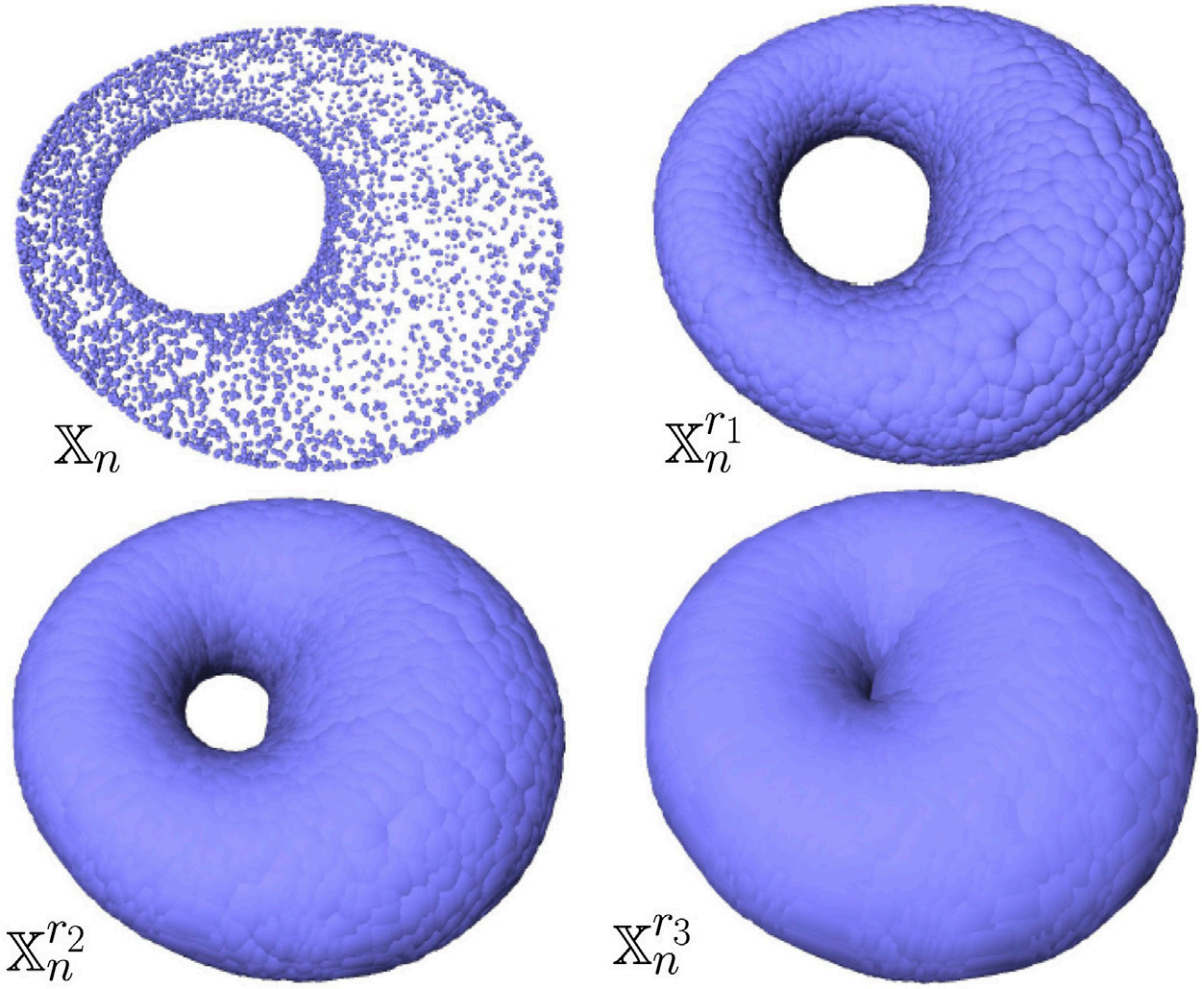
Example of a point cloud $X_{n}$ sampled on the surface of a torus in $R^{3}$ (top left) and its offsets for different values of radii *r*_1_ < *r*_2_ < *r*_3_. For well-chosen values of the radius (e.g., *r*_1_ and *r*_2_), the offsets are clearly homotopy equivalent to a torus.

In our setting, the considered compact sets are the data set $X_{n}$ and of the support *M* of the measure *μ*. When *M* is a smooth compact submanifold, under mild conditions on $d_{H}\left(X_{n},M\right)$ , for some well-chosen *r*, the offsets of $X_{n}$ are homotopy equivalent to *M* Chazal and Lieutier (2008), Niyogi et al. (2008) (see Figure 5 for an illustration). These results extend to larger classes of compact sets and lead to stronger results on the inference of the isotopy type of the offsets of *M* Chazal et al. (2009c), Chazal et al. (2009d). They also lead to results on the estimation of other geometric and differential quantities such as normals Chazal et al. (2009c), curvatures Chazal et al. (2009e), or boundary measures Chazal et al. (2010) under assumptions on the Hausdorff distance between the underlying shape and the data sample.

These results rely on the one-semiconcavity of the squared distance function $d_{K}^{2}$ , that is, the convexity of the function $x\to \|x{\|}^{2}-d_{K}^{2}\left(x\right)$ , and can be naturally stated in the following general framework.

**Definition 4.** *A function* $\phi :R^{d}\to R_{+}$ *is distance-like if it is proper (the preimage of any compact set in* R *is a compact set in* $R^{d}$ *) and x* → ‖*x*‖^2^ − *ϕ* ^2^(*x*) *is convex.*

Thanks to its semiconcavity, a distance-like function *ϕ* has a well-defined, but not continuous, gradient $\nabla \phi :R^{d}\to R^{d}$ that can be integrated into a continuous flow (Petrunin, 2007) that allows us to track the evolution of the topology of its sublevel sets and to compare it to one of the sublevel sets of close distance-like functions.

**Definition 5.** *Let ϕ be a distance-like function and let ϕ* ^*r*^ = *ϕ* ^−1^([0, *r*]) *be the r-sublevel set of ϕ.*

• *A point* $x\in R^{d}$ *is called α-critical if* ‖∇_*x*_ *ϕ*‖ ≤ *α. The corresponding value r* = *ϕ*(*x*) *is also said to be α-critical.*
• *The weak feature size of ϕ at r is the minimum r*′ > 0 *such that ϕ does not have any critical value between r and r* + *r*′*. We denote it by* wfs_*ϕ*_(*r*)*. For any* 0 < *α* < 1*, the α-reach of ϕ is the maximum r such that ϕ* ^−1^((0, *r*]) *does not contain any α-critical point.*

The weak feature size wfs_*ϕ*_(*r*) (resp. *α*-reach) measures the regularity of *ϕ* around its *r*-level sets (resp. *O*-level set). When *ϕ* = *d*_*K*_ is the distance function to a compact set $K\subset R^{d}$ , the one-reach coincides with the classical reach from geometric measure theory Federer (1959). Its estimation from random samples has been studied by Aamari et al. (2019). An important property of a distance-like function *ϕ* is that the topology of their sublevel sets *ϕ* ^*r*^ can only change when *r* crosses a 0-critical value.

**Lemma 1** (isotopy lemma grove (1993)). *Let ϕ be a distance-like function and r*_1_ < *r*_2_ *be two positive numbers such that ϕ has no 0-critical point, that is, points x such that* ∇*ϕ*(*x*) = 0*, in the subset ϕ* ^−1^([*r*_1_, *r*_2_])*. Then all the sublevel sets ϕ* ^−1^([0, *r*]) *are isotopic for r* ∈ [*r*_1_, *r*_2_]*.*

As an immediate consequence of the isotopy lemma, all the sublevel sets of *ϕ* between *r* and *r* + wfs_*ϕ*_(*r*) have the same topology. Now the following reconstruction theorem from Chazal et al. (2011b) provides a connection between the topology of the sublevel sets of close distance-like functions.

**Theorem 2** (Reconstruction theorem). *Let ϕ*, *ψ be two distance-like functions such that* ‖*ϕ* − *ψ*‖_*∞*_ < *ɛ, with* reach_*α*_(*ϕ*) ≥ *R for some positive ɛ and α. Then, for every r* ∈ [4*ɛ*/*α* ^2^, *R* − 3*ɛ*] *and every η* ∈ (0, *R*)*, the sublevel sets ψ* ^*r*^ *and ϕ* ^*η*^ *are homotopy equivalent when*

$$\varepsilon \leq \frac{R}{5+4/\alpha^{2}}.$$

Under similar but slightly more technical conditions, the Reconstruction theorem can be extended to prove that the sublevel sets are indeed homeomorphic and even isotopic (Chazal et al., 2009c; Chazal et al., 2008).

Coming back to our setting and taking for *ϕ* = *d*_*M*_ and $\psi =d_{X_{n}}$ the distance functions to the support *M* of the measure *μ* and to the data set $X_{n}$ , the condition reach_*α*_(*d*_*M*_) ≥ *R* can be interpreted as the regularity condition on *M* ^6^. The Reconstruction theorem combined with the Nerve theorem tells that for well-chosen values of *r*, *η* and the *η*-offsets of *M* are homotopy equivalent to the nerve of the union of balls of radius *r* centered on $X_{n}$ , that is, the Cech complex ${\mathrm{Cech}}_{r}\left(X_{n}\right)$ .

From a statistical perspective, the main advantage of these results involving the Hausdorff distance is that the estimation of the considered topological quantities boils down to support estimation questions that have been widely studied (see Section 4.3).

### 4.2 Homology Inference

The above results provide a mathematically well-founded framework to infer the topology of shapes from a simplicial complex built on the top of an approximating finite sample. However, from a more practical perspective, it raises two issues. First, the Reconstruction theorem requires a regularity assumption through the *α*-reach condition that may not always be satisfied and the choice of a radius *r* for the ball used to build the Čech complex ${\mathrm{Cech}}_{r}\left(X_{n}\right)$ . Second, ${\mathrm{Cech}}_{r}\left(X_{n}\right)$ provides a topologically faithful summary of the data through a simplicial complex that is usually not well suited for further data processing. One often needs topological descriptors that are easier to handle, in particular numerical ones, which can be easily computed from the complex. This second issue is addressed by considering the homology of the considered simplicial complexes in the next paragraph, while the first issue will be addressed in the next section with the introduction of persistent homology.

#### Homology in a Nutshell

Homology is a classical concept in algebraic topology, providing a powerful tool to formalize and handle the notion of the topological features of a topological space or of a simplicial complex in an algebraic way. For any dimension *k*, the *k*-dimensional “holes” are represented by a vector space *H*_*k*_, whose dimension is intuitively the number of such independent features. For example, the zero-dimensional homology group *H*_0_ represents the connected components of the complex, the one-dimensional homology group *H*_1_ represents the one-dimensional loops, the two-dimensional homology group *H*_2_ represents the two-dimensional cavities, and so on.

To avoid technical subtleties and difficulties, we restrict the introduction of homology to the minimum that is necessary to understand its usage in the following of the article. In particular, we restrict our information to homology with coefficients in $Z_{2}$ , that is, the field with two elements, 0 and 1, such that 1 + 1 = 0, which turns out to be geometrically a little bit more intuitive. However, all the notions and results presented in the sequel naturally extend to homology with coefficients in any field. We refer the reader to the study by Hatcher (2001) for a complete and comprehensible introduction to homology and to the study by Ghrist (2017) for a recent, concise, and very good introduction to applied algebraic topology and its connections to data analysis.

Let *K* be a (finite) simplicial complex and let *k* be a nonnegative integer. The space of *k*-chains on *K*, *C*_*k*_(*K*) is the set whose elements are the formal (finite) sums of *k*-simplices of *K*. More precisely, if {*σ*_1_, … , *σ*_*p*_} is the set of *k*-simplices of *K*, then any *k*-chain can be written as

$$c=\overset{p}{\underset{i=1}{\sum }}\varepsilon_{i}\sigma_{i}\;\;with\;\;\varepsilon_{i}\in Z_{2}.$$

If $c^{\prime }=\sum_{i=1}^{p}\varepsilon_{i}^{\prime }\sigma_{i}$ is another *k*-chain and $\lambda \in Z_{2}$ , the sum *c* + *c*′ is defined as $c+c^{\prime }=\sum_{i=1}^{p}\left(\varepsilon_{i}+\varepsilon_{i}^{\prime }\right)\sigma_{i}$ and the product *λ*.*c* is defined as $\lambda .c=\sum_{i=1}^{p}\left(\lambda .\varepsilon_{i}\right)\sigma_{i}$ , making *C*_*k*_(*K*) a vector space with coefficients in $Z_{2}$ . Since we are considering coefficients in $Z_{2}$ , geometrically, a *k*-chain can be seen as a finite collection of *k*-simplices and the sum of two *k*-chains as the symmetric difference of the two corresponding collections^7^.

The boundary of a *k*-simplex *σ* = [*v*_0_, … , *v*_*k*_] is the (*k* − 1)-chain

$$\partial_{k}\left(\sigma \right)=\overset{k}{\underset{i=0}{\sum }}{\left(-1\right)}^{i}\left[v_{0},\ldots ,{\hat{v}}_{i},\ldots ,v_{k}\right]$$

where $\left[v_{0},\ldots ,{\hat{v}}_{i},\ldots ,v_{k}\right]$ is the (*k* − 1)-simplex spanned by all the vertices except *v*_*i*_ ^8^. As the *k*-simplices form a basis of *C*_*k*_(*K*), *∂*_*k*_ extends as a linear map from *C*_*k*_(*K*) to *C*_*k*−1_(*K*) called the boundary operator. The kernel *Z*_*k*_(*K*) = {*c* ∈ *C*_*k*_(*K*): *∂*_*k*_ = 0} of *∂*_*k*_ is called the space of *k*-cycles of *K*, and the image *B*_*k*_(*K*) = {*c* ∈ *C*_*k*_(*K*): *∃c*′ ∈ *C*_*k*+1_(*K*), *∂*_*k*+1_(*c*′) = *c*} of *∂*_*k*+1_ is called the space of *k*-boundaries of *K*. The boundary operators satisfy the following fundamental property:

$$\partial_{k-1}{}^{\circ}\partial_{k}\equiv 0\;\;\text{for any}\;\;k\geq 1.$$

**FIGURE 6:**
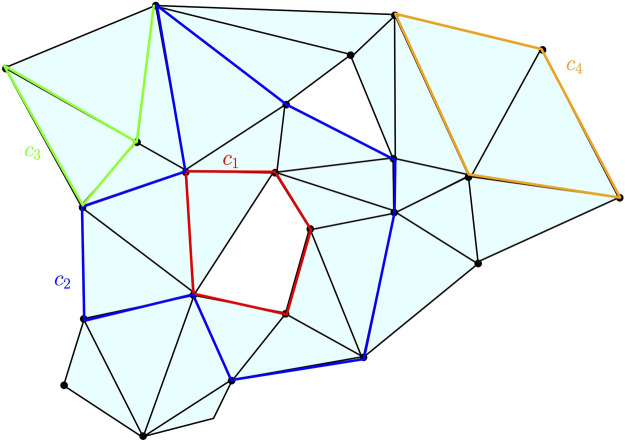
Some examples of chains, cycles, and boundaries on a two-dimensional complex *K*: *c*_1_, *c*_2_, and *c*_4_ are one-cycles; *c*_3_ is a one-chain but not a one-cycle; *c*_4_ is the one-boundary, namely, the boundary of the two-chain obtained as the sum of the two triangles surrounded by *c*_4_. The cycles *c*_1_ and *c*_2_ span the same element in *H*_1_(*K*) as their difference is the two-chain represented by the union of the triangles surrounded by the union of *c*_1_ and *c*_2_.

In other words, any *k*-boundary is a *k*-cycle, that is, *B*_*k*_(*K*) ⊆ *Z*_*k*_(*K*) ⊆ *C*_*k*_(*K*). These notions are illustrated in Figure 6.

**Definition 6** (simplicial homology group and Betti numbers)**.** *The k*th *(simplicial) homology group of K is the quotient vector space*

$$H_{k}\left(K\right)=Z_{k}\left(K\right)/B_{k}\left(K\right).$$

*The k*th *Betti number of K is the dimension β*_*k*_(*K*) = dim *H*_*k*_(*K*) *of the vector space H*_*k*_(*K*)*.*

**FIGURE 7:**
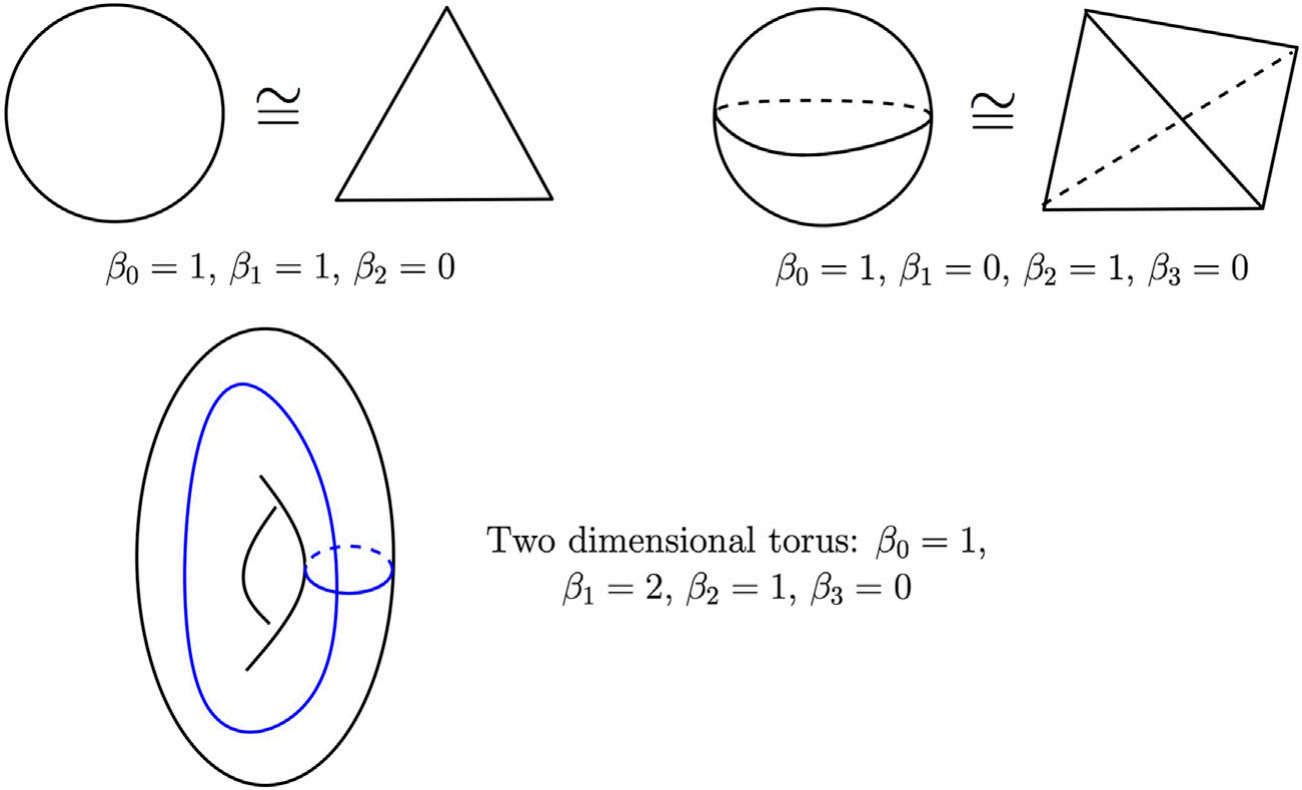
Betti numbers of the circle (top left), the two-dimensional sphere (top right), and the two-dimensional torus (bottom). The blue curves on the torus represent two independent cycles whose homology class is a basis of its one-dimensional homology group.

Figure 7 gives the Betti numbers of several simple spaces. Two cycles, *c*, *c*′ ∈ *Z*_*k*_(*K*), are said to be homologous if they differ by a boundary, that is, if there exists a (*k* + 1)-chain *d* such that *c*′ = *c* + *∂*_*k*+1_(*d*). Two such cycles give rise to the same element of *H*_*k*_. In other words, the elements of *H*_*k*_(*K*) are the equivalence classes of homologous cycles.

Simplicial homology groups and Betti numbers are topological invariants; if *K*, *K*′ are two simplicial complexes whose geometric realizations are homotopy equivalent, then their homology groups are isomorphic and their Betti numbers are the same.

Singular homology is another notion of homology that allows us to consider larger classes of topological spaces. It is defined for any topological space *X* similarly to simplicial homology, except that the notion of the simplex is replaced by the notion of the singular simplex, which is just any continuous map *σ*: *Δ*_*k*_ → *X* where *Δ*_*k*_ is the standard *k*-dimensional simplex. The space of *k*-chains is the vector space spanned by the *k*-dimensional singular simplices, and the boundary of a simplex *σ* is defined as the (alternated) sum of the restriction of *σ* to the (*k* − 1)-dimensional faces of *Δ*_*k*_. A remarkable fact about singular homology is that it coincides with simplicial homology whenever *X* is homeomorphic to the geometric realization of a simplicial complex. This allows us, in the sequel of this article, to indifferently talk about simplicial or singular homology for topological spaces and simplicial complexes.

Observing that if *f*: *X* → *Y* is a continuous map, then for any singular simplex *σ*: *Δ*_*k*_ → *X* in *X*, *f* °*σ*: *Δ*_*k*_ → *Y* is a singular simplex in *Y*, one easily deduces that continuous maps between topological spaces canonically induce homomorphisms between their homology groups. In particular, if *f* is a homeomorphism or a homotopy equivalence, then it induces an isomorphism between *H*_*k*_(*X*) and *H*_*k*_(*Y*) for any nonnegative integer *k*. As an example, it follows from the Nerve theorem that for any set of points $X\subset R^{d}$ and any *r* > 0, the *r*-offset *X* ^*r*^ and the Čech complex *Cech*_*r*_(*X*) have isomorphic homology groups and the same Betti numbers.

As a consequence, the Reconstruction theorem 2 leads to the following result on the estimation of Betti numbers.

**Theorem 3.** *Let* $M\subset R^{d}$ *be a compact set such that* reach_*α*_(*d*_*M*_) ≥ *R* > 0 *for some α* ∈ (0, 1) *and let* X *be a finite set of points such that* $d_{H}\left(M,X\right)=\varepsilon <\frac{R}{5+4/\alpha^{2}}$ *. Then, for every r* ∈ [4*ɛ*/*α* ^2^, *R* − 3*ɛ*] *and every η* ∈ (0, *R*)*, the Betti numbers of* ${\mathrm{Cech}}_{r}\left(X\right)$ *are the same as the ones of M* ^*η*^ *.*

*In particular, if M is a smooth m-dimensional submanifold of* $R^{d}$ *, then* $\beta_{k}\left({\mathrm{Cech}}_{r}\left(X\right)\right)=\beta_{k}\left(M\right)$ *for any k* = 0, … , *m.*

From a practical perspective, this result raises three difficulties: first, the regularity assumption involving the *α*-reach of *M* may be too restrictive; second, the computation of the nerve of a union of balls requires the use of a tricky predicate testing the emptiness of a finite union of balls; third, the estimation of the Betti numbers relies on the scale parameter *r*, whose choice may be a problem.

To overcome these issues, Chazal and Oudot (2008) established the following result, which offers a solution to the first two problems.

**Theorem 4.** *Let* $M\subset R^{d}$ *be a compact set such that* $wfs\left(M\right)={wfs}_{d_{M}}\left(0\right)\geq R>0$ *and let* X *be a finite set of points such that* $d_{H}\left(M,X\right)=\varepsilon <\frac{1}{9}wfs\left(M\right)$ *. Then for any* $r\in \left[2\varepsilon ,\frac{1}{4}\left(wfs\left(M\right)-\varepsilon \right)\right]$ *and any η* ∈ (0, *R*)*,*

$$\beta_{k}\left(X^{\eta }\right)=rk\left(H_{k}\left({\mathrm{Rips}}_{r}\left(X\right)\right)\to H_{k}\left({\mathrm{Rips}}_{4r}\left(X\right)\right)\right)$$

*where rk* $\left(H_{k}\left({\mathrm{Rips}}_{r}\left(X\right)\right)\to H_{k}\left({\mathrm{Rips}}_{4r}\left(X\right)\right)\right)$ *denotes the rank of the homomorphism induced by the (continuous) canonical inclusion* ${\mathrm{Rips}}_{r}\left(X\right)↪{\mathrm{Rips}}_{4r}\left(X\right)$ *.*

Although this result leaves the question of the choice of the scale parameter *r* open, it is proven in the study by Chazal and Oudot (2008) that a multiscale strategy whose description is beyond the scope of this article provides some help in identifying the relevant scales on which Theorem 4 can be applied.

### 4.3 Statistical Aspects of Homology Inference

According to the stability results presented in the previous section, a statistical approach to topological inference is strongly related to the problem of distribution support estimation and level sets estimation under the Hausdorff metric. A large number of methods and results are available for estimating the support of a distribution in statistics. For instance, the Devroye and Wise estimator (Devroye and Wise, 1980) defined on a sample $X_{n}$ is also a particular offset of $X_{n}$ . The convergence rates of both $X_{n}$ and the Devroye and Wise estimator to the support of the distribution for the Hausdorff distance were studied by Cuevas and Rodríguez-Casal (2004) in $R^{d}$ . More recently, the minimax rates of convergence of manifold estimation for the Hausdorff metric, which is particularly relevant for topological inference, has been studied by Genovese et al. (2012). There is also a large body of literature about level sets estimation in various metrics (see, for instance, Cadre, 2006; Polonik, 1995; Tsybakov, 1997) and, more particularly, for the Hausdorff metric Chen et al. (2017). All these works about support and level sets estimation shed light on the statistical analysis of topological inference procedures.

In the study by Niyogi et al. (2008), it was shown that the homotopy type of Riemannian manifolds with a reach larger than a given constant can be recovered with high probability from offsets of a sample on (or close to) the manifold. This article was probably the first attempt to consider the topological inference problem in terms of probability. The result of the study by Niyogi et al. (2008) was derived from a retract contraction argument and was on tight bounds over the packing number of the manifold in order to control the Hausdorff distance between the manifold and the observed point cloud. The homology inference in the noisy case, in the sense that the distribution of the observation is concentrated around the manifold, was also studied by Niyogi et al. (2008), Niyogi et al. (2011). The assumption that the geometric object is a smooth Riemannian manifold is only used in the article to control in probability the Hausdorff distance between the sample and the manifold and is not actually necessary for the “topological part” of the result. Regarding the topological results, these are similar to those of the studies by Chazal et al. (2009d), Chazal and Lieutier (2008) in the particular framework of Riemannian manifolds. Starting from the result of the study by Niyogi et al. (2008), the minimax rates of convergence of the homology type have been studied by Balakrishna et al. (2012) under various models for Riemannian manifolds with a reach larger than a constant. In contrast, a statistical version of the work of Chazal et al. (2009d) has not yet been proposed.

More recently, following the ideas of Niyogi et al. (2008), Bobrowski et al. (2014) have proposed a robust homology estimator for the level sets of both density and regression functions, by considering the inclusion map between nested pairs of estimated level sets (in the spirit of Theorem 4 above) obtained using a plug-in approach from a kernel estimator.

### 4.4 Going Beyond Hausdorff Distance: Distance to Measure

**FIGURE 8:**
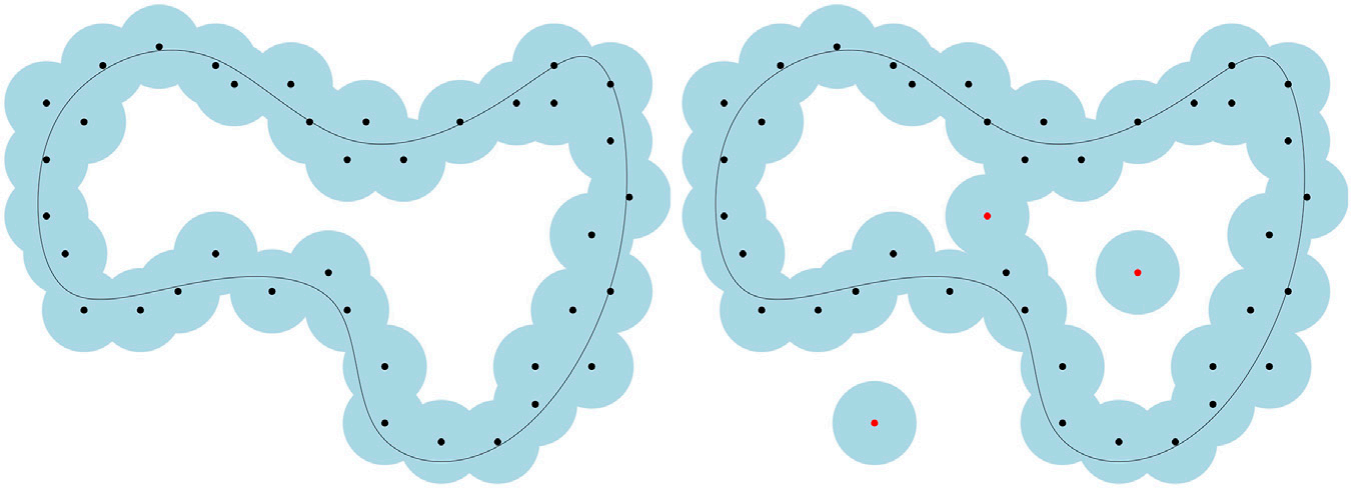
Effect of outliers on the sublevel sets of distance functions. Adding just a few outliers to a point cloud may dramatically change its distance function and the topology of its offsets.

It is well known that distance-based methods in tda may fail completely in the presence of outliers. Indeed, adding even a single outlier to the point cloud can change the distance function dramatically (see Figure 8 for an illustration). To answer this drawback, Chazal et al. (2011b) have introduced an alternative distance function which is robust to noise, the distance-to-measure.

Given a probability distribution *P* in $R^{d}$ and a real parameter 0 ≤ *u* ≤ 1, the notion of distance to the support of *P* may be generalized as the function

$$\delta_{P,u}:x\in R^{d}\mapsto \inf\left\{t>0\;;\;P\left(B\left(x,t\right)\right)\geq u\right\},$$

where *B*(*x*, *t*) is the closed Euclidean ball of center *x* and radius *t*. To avoid issues due to discontinuities of the map *P* → *δ*_*P*,*u*_, the distance-to-measure (DTM) function with parameter *m* ∈ [0, 1] and power *r* ≥ 1 is defined by

$$d_{P,m,r}\left(x\right):x\in R^{d}\mapsto {\left(\frac{1}{m}\int_{0}^{m}\delta_{P,u}^{r}\left(x\right)du\right)}^{1/r}.$$ (1)

A nice property of the DTM proved by Chazal et al. (2011b) is its stability with respect to perturbations of *P* in the Wasserstein metric. More precisely, the map *P* → *d*_*P*,*m*,*r*_ is $m^{-\frac{1}{r}}$ -Lipschitz, that is, if *P* and $\tilde{P}$ are two probability distributions on $R^{d}$ , then

$$\|d_{P,m,r}-d_{\tilde{P},m,r}{\|}_{\infty }\leq m^{-\frac{1}{r}}W_{r}\left(P,\tilde{P}\right)$$ (2)

where *W*_*r*_ is the Wasserstein distance for the Euclidean metric on $R^{d}$ , with exponent *r* ^9^. This property implies that the DTM associated with close distributions in the Wasserstein metric have close sublevel sets. Moreover, when *r* = 2, the function $d_{P,m,2}^{2}$ is semiconcave, ensuring strong regularity properties on the geometry of its sublevel sets. Using these properties, Chazal et al. (2011b) showed that under general assumptions, if $\tilde{P}$ is a probability distribution approximating *P*, then the sublevel sets of $d_{\tilde{P},m,2}$ provide a topologically correct approximation of the support of *P*.

In practice, the measure *P* is usually only known through a finite set of observations $X_{n}=\left\{X_{1},\ldots ,X_{n}\right\}$ sampled from *P*, raising the question of the approximation of the DTM. A natural idea to estimate the DTM from $X_{n}$ is to plug the empirical measure *P*_*n*_ instead of *P* into the definition of the DTM. This “plug-in strategy” corresponds to computing the distance to the empirical measure (DTEM). For $m=\frac{k}{n}$ , the DTEM satisfies

$$d_{P_{n},k/n,r}^{r}\left(x\right)≔\frac{1}{k}\overset{k}{\underset{j=1}{\sum }}{\left\|x-X_{n}\right\|}_{\left(j\right)}^{r},$$

where $\|x-X_{n}{\|}_{\left(j\right)}$ denotes the distance between *x* and its *j*th neighbor in {*X*_1_, *…* , *X*_*n*_}. This quantity can be easily computed in practice since it only requires the distances between *x* and the sample points. The convergence of the DTEM to the DTM has been studied by Chazal et al. (2017) and Chazal et al. (2016b).

The introduction of the DTM has motivated further works and applications in various directions such as topological data analysis (Buchet et al., 2015a), GPS trace analysis (Chazal et al., 2011a), density estimation (Biau et al., 2011), hypothesis testing Brécheteau (2019), and clustering (Chazal et al., 2013), just to name a few. Approximations, generalizations, and variants of the DTM have also been considered (Guibas et al., 2013; Phillips et al., 2014; Buchet et al., 2015b; Brécheteau and Levrard, 2020).

## 5 Persistent Homology

Persistent homology is a powerful tool used to efficiently compute, study, and encode multiscale topological features of nested families of simplicial complexes and topological spaces. It does not only provide efficient algorithms to compute the Betti numbers of each complex in the considered families, as required for homology inference in the previous section, but also encodes the evolution of the homology groups of the nested complexes across the scales. Ideas and preliminary results underlying persistent homology theory can be traced back to the 20th century, in particular in the works of Barannikov (1994), Frosini (1992), Robins (1999). It started to know an important development in its modern form after the seminal works of Edelsbrunner et al. (2002) and Zomorodian and Carlsson (2005).

### 5.1 Filtrations

A filtration of a simplicial complex *K* is a nested family of subcomplexes ${\left(K_{r}\right)}_{r\in T}$ , where T⊆R , such that for any *r*, *r*′ ∈ *T*, if *r* ≤ *r*′ then *K*_*r*_ ⊆ *K*_*r*’_ and *K* = ∪_*r*∈*T*_ *K*_*r*_. The subset *T* may be either finite or infinite. More generally, a filtration of a topological space M is a nested family of subspaces ${\left(M_{r}\right)}_{r\in T}$ , where T⊆R , such that for any *r*, *r*′ ∈ *T*, if *r* ≤ *r*′ then *M*_*r*_ ⊆ *M*_*r*’_ and *M* = ∪_*r*∈*T*_ *M*_*r*_. For example, if f:M→R is a function, then the family *M*_*r*_ = *f* ^−1^((−*∞*, *r*]), r∈R defines a filtration called the sublevel set filtration of *f*.

In practical situations, the parameter *r* ∈ *T* can often be interpreted as a scale parameter, and filtrations classically used in TDA often belong to one of the two following families.

### Filtrations Built on Top of Data

Given a subset X of a compact metric space (*M*, *ρ*), the families of Rips–Vietoris complexes ${\left({\mathrm{Rips}}_{r}\left(X\right)\right)}_{r\in R}$ and Čech complexes ${\left({\mathrm{Cech}}_{r}\left(X\right)\right)}_{r\in R}$ are filtrations^10^. Here, the parameter *r* can be interpreted as a resolution at which one considers the data set X . For example, if X is a point cloud in $R^{d}$ , thanks to the Nerve theorem, the filtration ${\left({\mathrm{Cech}}_{r}\left(X\right)\right)}_{r\in R}$ encodes the topology of the whole family of unions of balls $X^{r}=\cup_{x\in X}B\left(x,r\right)$ , as *r* goes from 0 to + *∞*. As the notion of filtration is quite flexible, many other filtrations have been considered in the literature and can be constructed on the top of data, such as the so-called witness complex popularized in tda by De Silva and Carlsson (2004), the weighted Rips filtrations Buchet et al. (2015b), or the so-called DTM filtrations Anai et al. (2019) that allow us to handle data corrupted by noise and outliers.

### Sublevel Sets Filtrations

Functions defined on the vertices of a simplicial complex give rise to another important example of filtration: let *K* be a simplicial complex with vertex set *V* and f:V→R . Then *f* can be extended to all simplices of *K* by *f*([*v*_0_, … , *v*_*k*_]) = max{*f*(*v*_*i*_): *i* = 1, … , *k*} for any simplex *σ* = [*v*_0_, … , *v*_*k*_] ∈ *K* and the family of subcomplexes, *K*_*r*_ = {*σ* ∈ *K*: *f*(*σ*) ≤ *r*}, defines a filtration called the sublevel set filtration of *f*. Similarly, one can define the upper-level set filtration of *f*.

In practice, even if the index set is infinite, all the considered filtrations are built on finite sets and are indeed finite. For example, when X is finite, the Vietoris–Rips complex ${\mathrm{Rips}}_{r}\left(X\right)$ changes only at a finite number of indices, *r*. This allows us to easily handle them from an algorithmic perspective.

### 5.2 Starting With a Few Examples

Given a filtration $\mathrm{Filt}={\left(F_{r}\right)}_{r\in T}$ of a simplicial complex or a topological space, the homology of *F*_*r*_ changes as *r* increases; new connected components can appear, existing components can merge, loops and cavities can appear or be filled, etc. Persistent homology tracks these changes, identifies the appearing features, and associates a lifetime with them. The resulting information is encoded as a set of intervals called a barcode or, equivalently, as a multiset of points in $R^{2}$ where the coordinate of each point is the starting and end point of the corresponding interval.

Before giving formal definitions, we introduce and illustrate persistent homology on a few simple examples.

### Example 1

**FIGURE 9:**
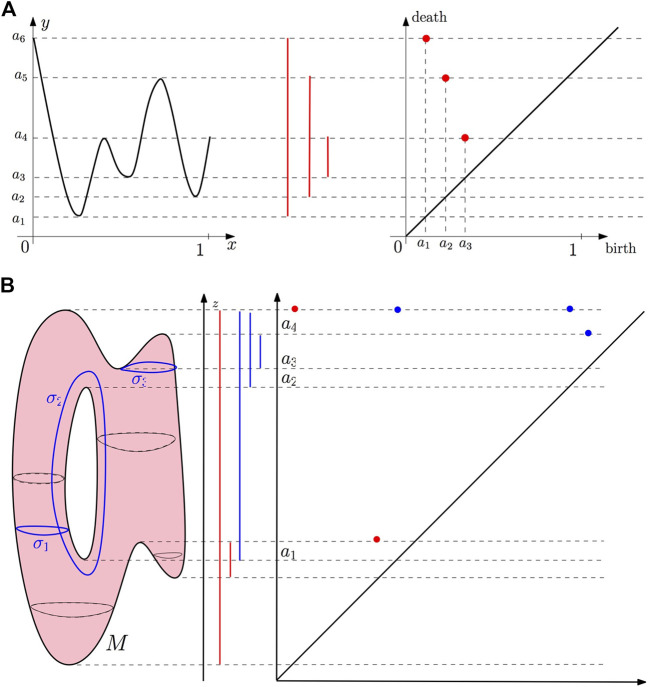
**(A)** Example 1: the persistence barcode and the persistence diagram of a function f:[0,1]→R . **(B)** Example 2: the persistence barcode and the persistence diagram of the height function (projection on the *z*-axis) defined on a surface in $R^{3}$ .

Let f:[0,1]→R be the function of Figure 9A and let $\left(\right.F_{r}=f^{-1}{\left(\left(-\infty ,r\left.\right]\right)\right)}_{r\in R}$ be the sublevel set filtration of *f*. All the sublevel sets of *f* are either empty or a union of intervals, so the only nontrivial topological information they carry is their zero-dimensional homology, that is, their number of connected components. For *r* < *a*_1_, *F*_*r*_ is empty, but at *r* = *a*_1_, a first connected component appears in $F_{a_{1}}$ . Persistent homology thus registers *a*_1_ as the birth time of a connected component and starts to keep track of it by creating an interval starting at *a*_1_. Then, *F*_*r*_ remains connected until *r* reaches the value *a*_2_, where a second connected component appears. Persistent homology starts to keep track of this new connected component by creating a second interval starting at *a*_2_. Similarly, when *r* reaches *a*_3_, a new connected component appears and persistent homology creates a new interval starting at *a*_3_. When *r* reaches *a*_4_, the two connected components created at *a*_1_ and *a*_3_ merge together to give a single larger component. At this step, persistent homology follows the rule that it is the most recently appeared component in the filtration that dies; the interval started at *a*_3_ is thus ended at *a*_4_, and a first persistence interval encoding the life span of the component born at *a*_3_ is created. When *r* reaches *a*_5_, as in the previous case, the component born at *a*_2_ dies, and the persistent interval (*a*_2_, *a*_5_) is created. The interval created at *a*_1_ remains until the end of the filtration, giving rise to the persistent interval (*a*_1_, *a*_6_), if the filtration is stopped at *a*_6_, or (*a*_1_, + *∞*), if *r* goes to + *∞* (notice that in this latter case, the filtration remains constant for *r* > *a*_6_). The obtained set of intervals encoding the life span of the different homological features encountered along the filtration is called the persistence barcode of *f*. Each interval (*a*, *a*′) can be represented by the point of coordinates (*a*, *a*′) in the $R^{2}$ plane. The resulting set of points is called the persistence diagram of *f*. Notice that a function may have several copies of the same interval in its persistence barcode. As a consequence, the persistence diagram of *f* is indeed a multi-set where each point has an integer-valued multiplicity. Last, for technical reasons that will become clear in the next section, one adds to the persistence all the points of the diagonal Δ = {(*b*, *d*): *b* = *d*} with an infinite multiplicity.

### Example 2

Let f:M→R now be the function of Figure 9B, where *M* is a two-dimensional surface homeomorphic to a torus, and let $\left(\right.F_{r}=f^{-1}{\left(\left(-\infty ,r\left.\right]\right)\right)}_{r\in R}$ be the sublevel set filtration of *f*. The zero-dimensional persistent homology is computed as in the previous example, giving rise to the red bars in the barcode. Now, the sublevel sets also carry one-dimensional homological features. When *r* goes through the height *a*_1_, the sublevel sets *F*_*r*_ that were homeomorphic to two discs become homeomorphic to the disjoint union of a disc and an annulus, creating a first cycle homologous to *σ*_1_ in Figure 9B. An interval (in blue) representing the birth of this new one-cycle is thus started at *a*_1_. Similarly, when *r* goes through the height *a*_2_, a second cycle, homologous to *σ*_2_, is created, giving rise to the start of a new persistent interval. These two created cycles are never filled (indeed, they span *H*_1_(*M*)) and the corresponding intervals remain until the end of the filtration. When *r* reaches *a*_3_, a new cycle is created that is filled and thus dies at *a*_4_, giving rise to the persistence interval (*a*_3_, *a*_4_). So now, the sublevel set filtration of *f* gives rise to two barcodes, one for zero-dimensional homology (in red) and one for one-dimensional homology (in blue). As previously stated, these two barcodes can equivalently be represented as diagrams in the plane.

### Example 3

**FIGURE 10:**
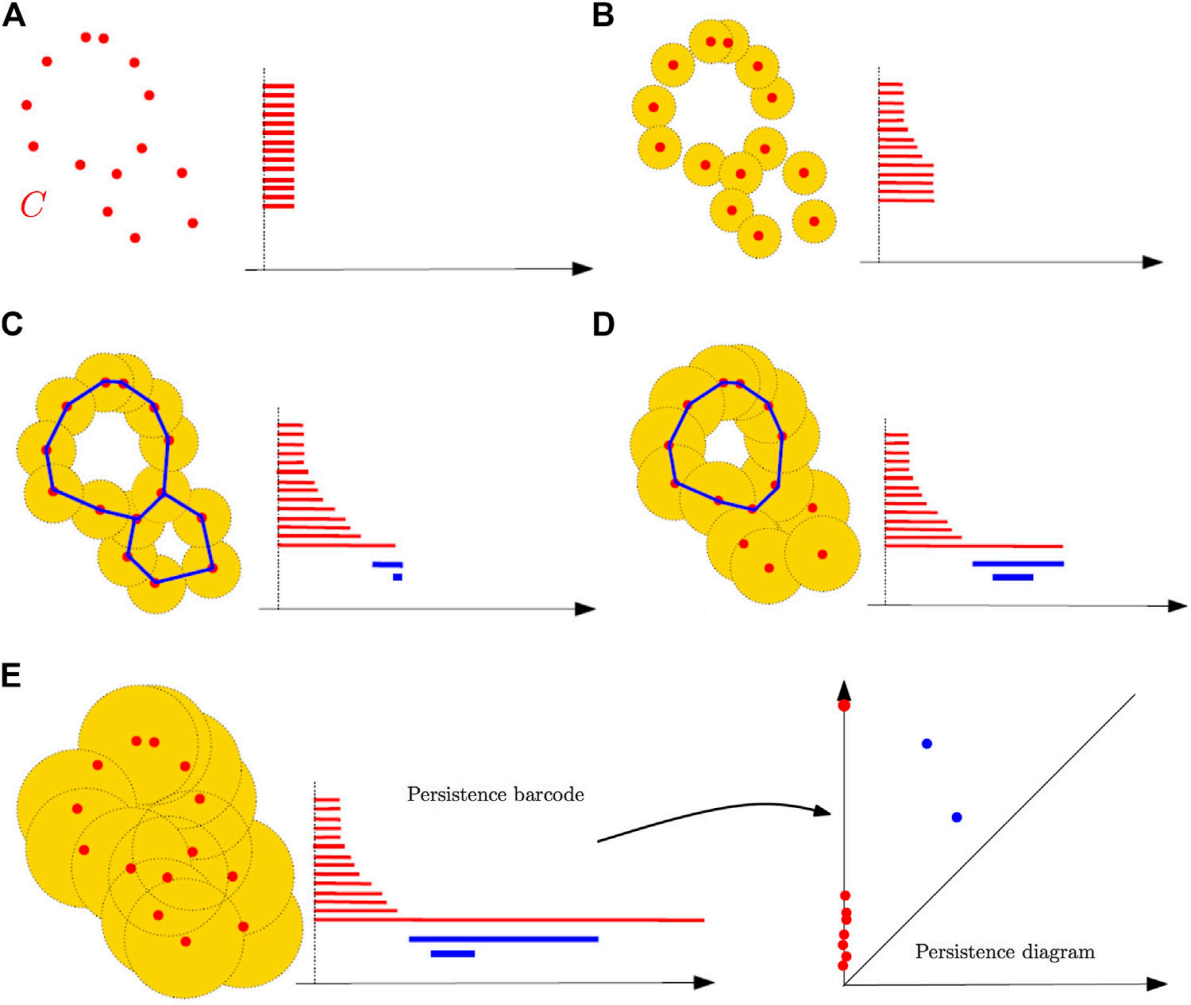
The sublevel set filtration of the distance function to a point cloud and the construction of its persistence barcode as the radius of balls increases. The blue curves in the unions of balls represent one-cycles associated with the blue bars in the barcodes. The persistence diagram is finally defined from the persistence barcodes. **(A)** For the radius *r* = 0, the union of balls is reduced to the initial finite set of points, each of them corresponding to a zero-dimensional feature, that is, a connected component; an interval is created for the birth for each of these features at *r* = 0. **(B)** Some of the balls started to overlap, resulting in the death of some connected components that get merged together; the persistence diagram keeps track of these deaths, putting an end point to the corresponding intervals as they disappear. **(C)** New components have merged, giving rise to a single connected component and, so, all the intervals associated with a zero-dimensional feature have been ended, except the one corresponding to the remaining components; two new one-dimensional features have appeared, resulting in two new intervals (in blue) starting on their birth scale. **(D)** One of the two one-dimensional cycles has been filled, resulting in its death in the filtration and the end of the corresponding blue interval. **(E)** All the one-dimensional features have died; only the long (and never dying) red interval remains. As in the previous examples, the final barcode can also be equivalently represented as a persistence diagram where every interval (*a*, *b*) is represented by the point of coordinates (*a*, *b*) in $R^{2}$ . Intuitively, the longer an interval in the barcode or, equivalently, the farther from the diagonal the corresponding point in the diagram, the more persistent, and thus relevant, the corresponding homological feature across the filtration. Notice also that for a given radius *r*, the *k*th Betti number of the corresponding union of balls is equal to the number of persistence intervals corresponding to *k*-dimensional homological features and containing *r.* So, the persistence diagram can be seen as a multiscale topological signature encoding the homology of the union of balls for all radii as well as its evolution across the values of *r.*

In this last example, we consider the filtration given by a union of growing balls centered on the finite set of points *C* in Figure 10. Notice that this is the sublevel set filtration of the distance function to *C*, and thanks to the Nerve theorem, this filtration is homotopy equivalent to the Čech filtration built on the top of *C*. Figure 10 shows several level sets of the filtration as follows:

a) For the radius *r* = 0, the union of balls is reduced to the initial finite set of points, each of them corresponding to a zero-dimensional feature, that is, a connected component; an interval is created for the birth for each of these features at *r* = 0.
b) Some of the balls started to overlap, resulting in the death of some connected components that get merged together; the persistence diagram keeps track of these deaths, putting an end point to the corresponding intervals as they disappear.
c) New components have merged, giving rise to a single connected component and, so, all the intervals associated with a zero-dimensional feature have been ended, except the one corresponding to the remaining components; two new one-dimensional features have appeared, resulting in two new intervals (in blue) starting on their birth scale.
d) One of the two one-dimensional cycles has been filled, resulting in its death in the filtration and the end of the corresponding blue interval.
e) All the one-dimensional features have died; only the long (and never dying) red interval remains. As in the previous examples, the final barcode can also be equivalently represented as a persistence diagram where every interval (*a*, *b*) is represented by the point of coordinates (*a*, *b*) in $R^{2}$ . Intuitively, the longer an interval in the barcode or, equivalently, the farther from the diagonal the corresponding point in the diagram, the more persistent, and thus relevant, the corresponding homological feature across the filtration. Notice also that for a given radius *r*, the *k*th Betti number of the corresponding union of balls is equal to the number of persistence intervals corresponding to *k*-dimensional homological features and containing *r.* So, the persistence diagram can be seen as a multiscale topological signature encoding the homology of the union of balls for all radii as well as its evolution across the values of *r.*

### 5.3 Persistent Modules and Persistence Diagrams

Persistent diagrams can be formally and rigorously defined in a purely algebraic way. This requires some care, and we only give the basic necessary notions here, leaving aside technical subtleties and difficulties. We refer the readers interested in a detailed exposition to Chazal et al. (2016a).

Let $\mathrm{Filt}={\left(F_{r}\right)}_{r\in T}$ be a filtration of a simplicial complex or a topological space. Given a nonnegative integer *k* and considering the homology groups *H*_*k*_(*F*_*r*_), we obtain a sequence of vector spaces where the inclusions *F*_*r*_ ⊂ *F*_*r*’_, *r* ≤ *r*′ induce linear maps between *H*_*k*_(*F*_*r*_) and *H*_*k*_(*F*_*r*’_). Such a sequence of vector spaces together with the linear maps connecting them is called a persistence module.

Definition 7. *A persistence module* V *over a subset T of the real numbers* R *is an indexed family of vector spaces* (*V*_*r*_∣*r* ∈ *T*) *and a doubly indexed family of linear maps* $\left(v_{s}^{r}:V_{r}\to V_{s}|r\leq s\right)$ *which satisfy the composition law* $v_{t}^{s}◦v_{s}^{r}=v_{t}^{r}$ *whenever r* ≤ *s* ≤ *t, and where* $v_{r}^{r}$ *is the identity map on V*_*r*_ *.*

In many cases, a persistence module can be decomposed into a direct sum of interval modules $I_{\left(b,d\right)}$ of the form

$$\ldots ,\to 0\to \ldots ,\to 0\to Z_{2}\to \ldots ,\to Z_{2}\to 0\to \ldots$$

where the maps $Z_{2}\to Z_{2}$ are identity maps while all the other maps are 0. Denoting *b* (resp. *d*), the infimum (resp. supremum) of the interval of indices corresponds to nonzero vector spaces; such a module can be interpreted as a feature that appears in the filtration at index *b* and disappears at index *d*. When a persistence module V can be decomposed as a direct sum of interval modules, one can show that this decomposition is unique up to reordering the intervals (see (Chazal et al., 2016a, Theorem 2.7)). As a consequence, the set of resulting intervals is independent of the decomposition of V and is called the persistence barcode of V . As in the examples of the previous section, each interval (*b*, *d*) in the barcode can be represented as the point of coordinates (*b*, *d*) in the plane $R^{2}$ . The disjoint union of these points, together with the diagonal Δ = {*x* = *y*}, is a multi-set called the persistence diagram of V .

The following result, from (Chazal et al., 2016a, Theorem 2.8), gives some necessary conditions for a persistence module to be decomposable as a direct sum of interval modules.

**Theorem 5.** *Let* V *be a persistence module indexed by* T⊂R *. If T is a finite set or if all the vector spaces V*_*r*_ *are finite-dimensional, then* V *is decomposable as a direct sum of interval modules. Moreover, for any s*, *t* ∈ *T, s* ≤ *t, the number* $\beta_{t}^{s}$ *of intervals starting before s and ending after t is equal to the rank of the linear map* $v_{t}^{s}$ *and is called the* (*s*, *t*)*-persistent Betti number of the filtration.*

As both conditions above are satisfied for the persistent homology of filtrations of finite simplicial complexes, an immediate consequence of this result is that the persistence diagrams of such filtrations are always well defined.

Indeed, it is possible to show that persistence diagrams can be defined as soon as the following simple condition is satisfied.

**Definition 8.** *A persistence module* V *indexed by* T⊂R *is q-tame if for any r* < *s in T, the rank of the linear map* $v_{s}^{r}:V_{r}\to V_{s}$ *is finite.*

**Theorem 6** Chazal et al. (2009a), Chazal et al. (2016a). *If* V *is a q-tame persistence module, then it has a well-defined persistence diagram. Such a persistence diagram* dgm(V) *is the union of the points of the diagonal* Δ *of* $R^{2}$ *, counted with infinite multiplicity, and a multi-set above the diagonal in* $R^{2}$ *that is locally finite. Here, by locally finite, we mean that for any rectangle R with sides parallel to the coordinate axes that does not intersect* Δ*, the number of points of* dgm(V) *, counted with multiplicity, contained in R is finite. Also, the part of the diagram made of the points with the infinite second coordinate is called the essential part of the diagram.*

The construction of persistence diagrams of q-tame modules is beyond the scope of this article, but it gives rise to the same notion as in the case of decomposable modules. It can be done either by following the algebraic approach based upon the decomposability properties of modules or by adopting a measure theoretic approach that allows us to define diagrams as integer-valued measures on a space of rectangles in the plane. We refer the reader to Chazal et al. (2016a) for more information.

Although persistence modules encountered in practice are decomposable, the general framework of the q-tame persistence module plays a fundamental role in the mathematical and statistical analysis of persistent homology. In particular, it is needed to ensure the existence of limit diagrams when convergence properties are studied (see Section 6).

A filtration $\mathrm{Filt}={\left(F_{r}\right)}_{r\in T}$ of a simplicial complex or of a topological space is said to be tame if for any integer *k*, the persistence module (*H*_*k*_(*F*_*r*_)∣*r* ∈ *T*) is *q*-tame. Notice that the filtrations of finite simplicial complexes are always tame. As a consequence, for any integer *k*, a persistence diagram denoted dgm_k_(Filt) is associated with the filtration Filt. When *k* is not explicitly specified and when there is no ambiguity, it is usual to drop the index *k* in the notation and to talk about “the” persistence diagram dgm(Filt) of the filtration Filt. This notation has to be understood as “dgm_k_(Filt) for some *k*.”

### 5.4 Persistence Landscapes

The persistence landscape introduced in the study by Bubenik (2015) is an alternative representation of persistence diagrams. This approach aims at representing the topological information encoded in persistence diagrams as elements of a Hilbert space, for which statistical learning methods can be directly applied. The persistence landscape is a collection of continuous, piecewise linear functions λ:N×R→R that summarizes a persistence diagram dgm.

**FIGURE 11:**
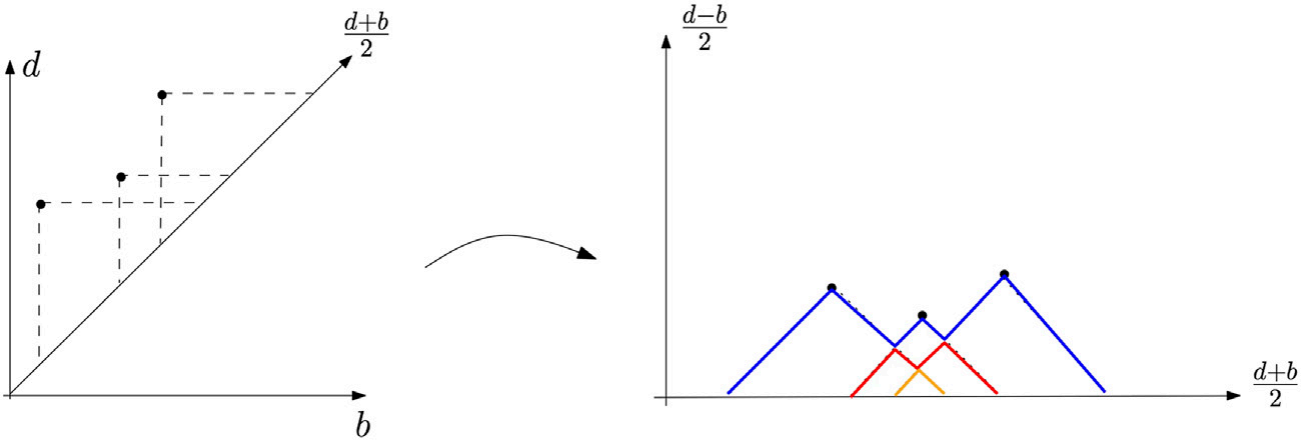
Example of a persistence landscape (right) associated with a persistence diagram (left). The first landscape is in blue, the second one in red, and the last one in orange. All the other landscapes are zero.

A birth–death pair *p* = (*b*, *d*) ∈ dgm is transformed into the point $\left(\frac{b+d}{2},\frac{d-b}{2}\right)$ (see Figure 11). Remember that the points with infinite persistence have been simply discarded in this definition. The landscape is then defined by considering the set of functions created by tenting the features of the rotated persistence diagram as follows:

$$\Lambda_{p}\left(t\right)=\left\{\begin{array}{cc} t-b\; & t\in \left[b,\frac{b+d}{2}\right]\\ d-t\; & t\in \left(\frac{b+d}{2},d\right]\\ 0\; & \text{otherwise}. \end{array}\right.$$ (3)

The persistence landscape *λ*_dgm_ of dgm is a summary of the arrangement of piecewise linear curves obtained by overlaying the graphs of the functions ${\left\{\Lambda_{p}\right\}}_{p\in \mathrm{dgm}}$ . Formally, the persistence landscape of dgm is the collection of functions

$$\lambda_{\mathrm{dgm}}\left(k,t\right)=\underset{r\in \mathrm{dgm}}{\text{kmax}}\;\Lambda_{r}\left(t\right),\;t\in \left[0,T\right],k\in N,$$ (4)

where kmax is the *k*th largest value in the set; in particular, 1max is the usual maximum function. Given k∈N , the function $\lambda_{\mathrm{dgm}}\left(k,.\right):R\to R$ is called the *k*th landscape of dgm. It is not difficult to see that the map that associates to each persistence diagram its corresponding landscape is injective. In other words, formally, no information is lost when a persistence diagram is represented through its persistence landscape.

The advantage of the persistence landscape representation is two-fold. First, persistence diagrams are mapped as elements of a functional space, opening the door to the use of a broad variety of statistical and data analysis tools for further processing of topological features see Bubenik (2015), Chazal et al. (2015c) and Section 6.3.1. Second, and fundamental from a theoretical perspective, the persistence landscapes share the same stability properties as those of persistence diagrams (see Section 5.7).

### 5.5 Linear Representations of Persistence Homology

A persistence diagram without its essential part can be represented as a discrete measure on Δ^+^ = {*p* = (*b*, *d*), *b* < *d* < *∞*}. With a slight abuse of notation, we can write the following:

$$\mathrm{dgm}=\underset{p\in \mathrm{dgm}}{\sum }\delta_{p},$$

where the features are counted with multiplicity and where *δ*_(*b*,*d*)_ denotes the Dirac measure in *p* = (*b*, *d*). Most of the persistence-based descriptors that have been proposed to analyze persistence can be expressed as linear transformations of the persistence diagram, seen as a point process

$$\Psi \left(\mathrm{dgm}\right)=\underset{p\in \mathrm{dgm}}{\sum }f\left(p\right),$$

for some function *f* defined on Δ and taking values in a Banach space.

In most cases, we want these transformations to apply independently at each homological dimension. For k∈N a given homological dimension, we then consider some linear transformation of the persistence diagram, restricted to the topological features of dimension *k* as follows:

$$\Psi_{k}\left({\mathrm{dgm}}_{k}\right)=\underset{p\in {\mathrm{dgm}}_{k}}{\sum }f_{k}\left(p\right),$$ (5)

where dgm_*k*_ is the persistence diagram of the topological features of dimension *k* and where *f*_*k*_ is defined on Δ and takes values in a Banach space.

### Betti Curve

The simplest way to represent persistence homology is the Betti function or the Betti curve. The Betti curve of homological dimension *k* is defined as

$$\beta_{k}\left(t\right)=\underset{\left(b,d\right)\in \mathrm{dgm}}{\sum }w\left(b,d\right)1_{t\in \left[b,d\right]}$$

where *w* is a weight function defined on Δ. In other words, the Betti curve is the number of barcodes at time *m*. This descriptor is a linear representation of persistence homology by taking *f* in (5) such that *f*(*b*, *d*) (*t*) = *w*(*b*, *d*)**1**_*t*∈[*b*,*d*]_. A typical choice for the weigh function is an increasing function of the persistence $w\left(b,d\right)=\tilde{w}\left(d-b\right)$ where $\tilde{w}$ is an increasing function defined on $R^{+}$ . One of the first applications of Betti curves can be found in the study by Umeda (2017).

### Persistence Surface

The persistence surface (also called persistence images) is obtained by making the convolution of a diagram with a kernel. It has been introduced in the study by Adams et al. (2017). For $K:R^{2}\to R$ , a kernel, and *H*, a 2 × 2 bandwidth matrix (e.g., a symmetric positive definite matrix), let for $u\in R^{2}$

$$K_{H}\left(u\right)=\det{\left(H\right)}^{-1/2}K\left(H^{-1/2}u\right).$$

Let $w:R^{2}\to R_{+}$ a weight function defined on Δ. One defines the persistence surface of homological dimension *k* associated with a diagram dgm, with kernel *K* and bandwidth matrix *H* by the following:

$$\forall u\in R^{2},\;\rho_{k}\left(\mathrm{dgm}\right)\left(u\right)=\underset{p\in {\mathrm{dgm}}_{k}}{\sum }w\left(r\right)K_{H}\left(u-p\right).$$

The persistence surface is obviously a linear representation of persistence homology. Typical weigh functions are increasing functions of the persistence.

### Other Linear Representations of Persistence

Many other linear representations of persistence have been proposed in the literature, such as the persistence silhouette (Chazal et al., 2015b), the accumulated persistence function (Biscio and Møller, 2019), and variants of the persistence surface (Reininghaus et al., 2015; Kusano et al., 2016; Chen et al., 2017).

Considering persistence diagrams as discrete measures and their vectorizations as linear representation is an approach that has also proven fruitful to studying distributions of diagrams Divol and Chazal (2020) and the metric structure of the space of persistence diagrams Divol and Lacombe (2020) (see Sections 5.6 and Section 6.3).

### 5.6 Metrics on the Space of Persistence Diagrams

To exploit the topological information and topological features inferred from persistent homology, one needs to be able to compare persistence diagrams, that is, to endow the space of persistence diagrams with a metric structure. Although several metrics can be considered, the most fundamental one is known as the bottleneck distance.

**FIGURE 12:**
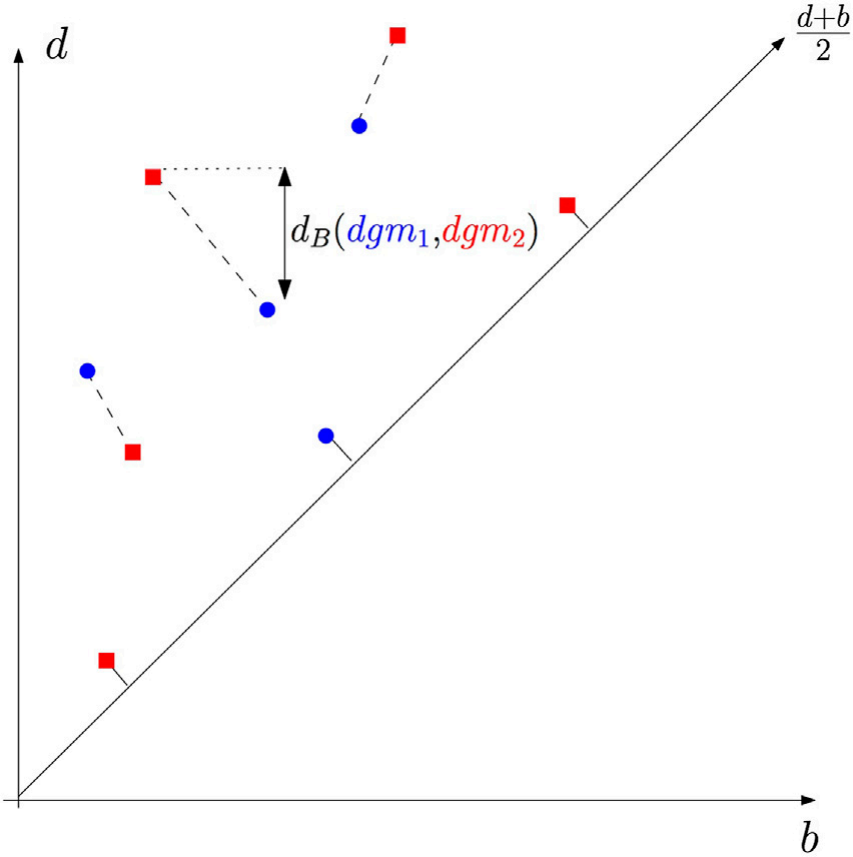
Perfect matching and the bottleneck distance between a blue and a red diagram. Notice that some points of both diagrams are matched to points of the diagonal.

Recall that a persistence diagram is the union of a discrete multi-set in the half-plane above the diagonal Δ and, for technical reasons that will become clear below, of Δ where the point of Δ is counted with infinite multiplicity. A matching (see Figure 12) between two diagrams, dgm_1_ and dgm_2_, is a subset *m* ⊆ dgm_1_ × dgm_2_ such that every point in dgm_1_ \Δ and dgm_2_ \Δ appears exactly once in *m*. In other words, for any *p* ∈ dgm_1_ \Δ and for any *q* ∈ dgm_2_ \Δ, ({*p*}× dgm_2_) ∩ *m* and (dgm_1_ ×{*q*}) ∩ *m* each contains a single pair. The bottleneck distance between dgm_1_ and dgm_2_ is then defined by

$$d_{b}\left({\mathrm{dgm}}_{1},{\mathrm{dgm}}_{2}\right)=\underset{\text{matching}\;m}{\inf}\underset{\left(p,q\right)\in m}{\max}\|p-q{\|}_{\infty }.$$

The practical computation of the bottleneck distance boils down to the computation of a perfect matching in a bipartite graph for which classical algorithms can be used.

The bottleneck metric is an *L*_*∞*_-like metric. It turns out to be the natural one to express stability properties of persistence diagrams presented in Section 5.7, but it suffers from the same drawbacks as the usual *L*_*∞*_ norms, that is, it is completely determined by the largest distance among the pairs and does not take into account the closeness of the remaining pairs of points. A variant to overcome this issue, the so-called Wasserstein distance between diagrams, is sometimes considered. Given *p* ≥ 1, it is defined by

$$W_{p}{\left({\mathrm{dgm}}_{1},{\mathrm{dgm}}_{2}\right)}^{p}=\underset{\text{matching}\;m}{\inf}\underset{\left(p,q\right)\in m}{\sum }\|p-q{\|}_{\infty }^{p}.$$

Useful stability results for persistence in the *W*_*p*_ metric exist among the literature, in particular the study by Cohen-Steiner et al. (2010), but they rely on assumptions that make them consequences of the stability results in the bottleneck metric. A general study of the space of persistence diagrams endowed with *W*_*p*_ metrics has been considered in the study by Divol and Lacombe (2020), where they proposed a general framework, based upon optimal partial transport, in which many important properties of persistence diagrams can be proven in a natural way.

### 5.7 Stability Properties of Persistence Diagrams

A fundamental property of persistence homology is that persistence diagrams of filtrations built on the top of data sets turn out to be very stable with respect to some perturbations of the data. To formalize and quantify such stability properties, we first need to be precise with regard to the notion of perturbation that is allowed.

Rather than working directly with filtrations built on the top of data sets, it turns out to be more convenient to define a notion of proximity between persistence modules, from which we will derive a general stability result for persistent homology. Then, most of the stability results for specific filtrations will appear as a consequence of this general theorem. To avoid technical discussions, from now on, we assume, without loss of generality, that the considered persistence modules are indexed by R .

**Definition 9.** *Let* V,W *be two persistence modules indexed by* R *. Given* δ∈R *, a homomorphism of degree δ between* V *and* W *is a collection* Φ *of linear maps ϕ*_*r*_: *V*_*r*_ → *W*_*r*+*δ*_ *, for all* r∈R *such that for any r* ≤ *s,* $\phi_{s}◦v_{s}^{r}=w_{s+\delta }^{r+\delta }◦\phi_{r}$ *.*

An important example of a homomorphism of degree *δ* is the shift endomorphism $1_{V}^{\delta }$ which consists of the families of linear maps $\left(v_{r+\delta }^{r}\right)$ . Notice also that homomorphisms of modules can naturally be composed; the composition of a homomorphism Ψ of degree *δ* between U and V and a homomorphism Φ of degree *δ*′ between V and W naturally gives rise to a homomorphism ΦΨ of degree *δ* + *δ*′ between U and W .

**Definition 10.** *Let δ* ≥ 0*. Two persistence modules* V,W *are δ-interleaved if there exist two homomorphisms of degree δ,* Φ*, from* V *to* W *and* Ψ*, from* W *to* V *such that* $\Psi \Phi =1_{V}^{2\delta }$ *and* $\Phi \Psi =1_{W}^{2\delta }$ *.*

Although it does not define a metric on the space of persistence modules, the notion of closeness between two persistence modules may be defined as the smallest nonnegative *δ* such that they are *δ*-interleaved. Moreover, it allows us to formalize the following fundamental theorem (Chazal et al., 2009a; Chazal et al., 2016a).

**Theorem 7** (Stability of persistence). *Let* V *and* W *be two q-tame persistence modules. If* V *and* W *are δ-interleaved for some δ* ≥ 0*, then*

$$d_{b}\left(\mathrm{dgm}\left(V\right),\mathrm{dgm}\left(W\right)\right)\leq \delta .$$

Although purely algebraic and rather abstract, this result is an efficient tool to easily establish concrete stability results in TDA. For example, we can easily recover the first persistence stability result that appeared in the literature (Cohen-Steiner et al., 2005).

**Theorem 8.** *Let* f,g:M→R *be two real-valued functions defined on a topological space M that are q-tame, that is, such that the sublevel set filtrations of f and g induce q-tame modules at the homology level. Then for any integer k,*

$$d_{b}\left({\mathrm{dgm}}_{k}\left(f\right),{\mathrm{dgm}}_{k}\left(g\right)\right)\leq \|f-g{\|}_{\infty }=\underset{x\in M}{\sup}|f\left(x\right)-g\left(x\right)|$$

*where* dgm_*k*_(*f*) *(resp.* dgm_*k*_(*g*)*) is the persistence diagram of the persistence module* $\left(H_{k}\left(f^{-1}\left(-\infty ,r\left.\right]\right)\right)|r\in R\right)$ *(resp.* $\left(H_{k}\left(g^{-1}\left(-\infty ,r\left.\right]\right)\right)|r\in R\right)$ *) where the linear maps are the one induced by the canonical inclusion maps between sublevel sets.*

Proof. Denoting δ = ‖f − g‖_∞_, we have that for any r∈R *,* $f^{-1}\left(-\infty ,r\left.\right]\right)\subseteq g^{-1}\left(-\infty ,r+\delta \left.\right]\right)$ and $g^{-1}\left(-\infty ,r\left.\right]\right)\subseteq f^{-1}\left(-\infty ,r+\delta \left.\right]\right)$ . This interleaving between the sublevel sets of f induces a δ-interleaving between the persistence modules at the homology level, and the result follows from the direct application of Theorem 7.

Theorem 7 also implies a stability result for the persistence diagrams of filtrations built on the top of data.

**Theorem 9.** *Let* X *and* Y *be two compact metric spaces and let* Filt(X) *and* Filt(Y) *be the Vietoris–Rips of Čech filtrations built on the top of* X *and* Y *. Then*

$$d_{b}\left(\mathrm{dgm}\left(\mathrm{Filt}\left(X\right)\right),\mathrm{dgm}\left(\mathrm{Filt}\left(Y\right)\right)\right)\leq 2d_{GH}\left(X,Y\right)$$

*where* dgm(Filt(X)) *and* dgm(Filt(Y)) *denote the persistence diagram of the filtrations* Filt(X) *and* Filt(X) *.*

As we already noticed in Example 3 of Section 5.2, the persistence diagrams can be interpreted as multiscale topological features of X and Y . In addition, Theorem 9 tells us that these features are robust with respect to perturbations of the data in the Gromov–Hausdorff metric. They can be used as discriminative features for classification or other tasks (see, for example, Chazal et al. (2009b) for an application to nonrigid 3D shape classification).

We now give similar results for the alternative persistence homology representations introduced before. From the definition of the persistence landscape, we immediately observe that λ(k, ⋅) is one-Lipschitz, and thus, stability properties similar to those for persistence diagrams are satisfied for the landscapes.

**Proposition 1** (stability of persistence landscapes; Bubenik (2015)). Let dgm and dgm’ be two persistence diagrams (without their essential parts). For any t∈R and any k∈N , we have the following:

(i) λ(*k*, *t*) ≥ λ(*k* + 1, *t*) ≥ 0.
(ii) $\left.|\lambda \left(k,t\right)-\lambda^{\prime }\left(k,t\right)|\;\leq \;d_{b}\left(\mathrm{dgm},{\mathrm{dgm}}^{\prime }\right)\right)$ .

A large class of linear representations is continuous with respect to the Wasserstein metric W_s_ in the space of persistence diagrams and with respect to the Banach norm of the linear representation of persistence. Generally speaking, it is not always possible to upper bound the modulus of continuity of the linear representation operator. However, in the case where s = 1, it is even possible to show a stability result if the weight function takes small values for points close to the diagonal (see Divol and Lacombe (2020), Hofer et al. (2019b)).

### Stability Versus Discriminative Capacity of Persistence Representations

The results of the study by Divol and Lacombe (2020) showed that continuity and stability are only possible with weigh functions taking small values for points close to the diagonal. However, in general, there is no specific reason to consider that points close to the diagonal are less important than others, given a learning task. In a machine learning perspective, it is also relevant to design linear representation with general weigh functions, although it would be more difficult to prove the consistency of the corresponding methods without at least the continuity of the representation. Stability is thus important but maybe too strong a requirement for many problems in data sciences. Designing linear representation that is sensitive to specific parts of persistence diagrams rather than globally stable may reveal a good strategy in practice.

## 6 Statistical Aspects of Persistent Homology

Persistence homology by itself does not take into account the random nature of data and the intrinsic variability of the topological quantity they infer. We now present a statistical approach to persistent homology, in the sense that data are considered to be generated from an unknown distribution. We start with several consistency results for persistent homology inference.

### 6.1 Consistency Results for Persistent Homology

Assume that we observe n points (X_1_, … , X_n_) in a metric space (M, ρ) drawn i. i. d. from an unknown probability measure μ whose support is a compact set denoted $X_{\mu }$ . The Gromov–Hausdorff distance allows us to compare $X_{\mu }$ with compact metric spaces not necessarily embedded in M. In the following, an estimator $\hat{X}$ of $X_{\mu }$ is a function of X_1_ … , X_n_ that takes values in the set of compact metric spaces.

Let $\mathrm{Filt}\left(X_{\mu }\right)$ and $\mathrm{Filt}\left(\hat{X}\right)$ be two filtrations defined on $X_{\mu }$ and $\hat{X}$ . Starting from Theorem 9; a natural strategy for estimating the persistent homology of $\mathrm{Filt}\left(X_{\mu }\right)$ consists in estimating the support $X_{\mu }$ . Note that in some cases, the space M can be unknown and the observations X_1_ … , X_n_ are then only known through their pairwise distances ρ(X_i_, X_j_), i, j = 1, … , n. The use of the Gromov–Hausdorff distance allows us to consider this set of observations as an abstract metric space of cardinality n, independently of the way it is embedded in M. This general framework includes the more standard approach consisting in estimating the support with respect to the Hausdorff distance by restraining the values of $\hat{X}$ to the compact sets included in M.

The finite set $X_{n}≔\left\{X_{1},\ldots ,X_{n}\right\}$ is a natural estimator of the support $X_{\mu }$ . In several contexts discussed in the following, $X_{n}$ shows optimal rates of convergence to $X_{\mu }$ with respect to the Hausdorff distance. For some constants a, b > 0, we say that μ satisfies the (a, b)-standard assumption if for any $x\in X_{\mu }$ and any r > 0,

$$\mu \left(B\left(x,r\right)\right)\geq \min\left(ar^{b},1\right).$$ (6)

This assumption has been widely used in the literature of set estimation under the Hausdorff distance (Cuevas and Rodríguez-Casal, 2004; Singh et al., 2009). Under this assumption, it can be easily derived that the rate of convergence of $\mathrm{dgm}\left(\mathrm{Filt}\left(X_{n}\right)\right)$ to $\mathrm{dgm}\left(\mathrm{Filt}\left(X_{\mu }\right)\right)$ for the bottleneck metric is upper bounded by $O{\left(\frac{\log n}{n}\right)}^{1/b}$ . More precisely, this rate upper bounds the minimax rate of convergence over the set of probability measures on the metric space (M, ρ) satisfying the (a, b)-standard assumption on M.

**Theorem 10.** *Chazal et al. (2014)* *For some positive constants a and b, let*

$$P≔\left\{\mu \;on\;M\;|\;X_{\mu }\;is\;compact\;and\;\forall x\in X_{\mu },\forall r>0,\;\mu \left(B\left(x,r\right)\right)\geq \min\left(1,ar^{b}\right)\right\}.$$

Then, it holds

$$\underset{\mu \in P}{\sup}E\left[d_{b}\left(\mathrm{dgm}\left(\mathrm{Filt}\left(X_{\mu }\right)\right),\mathrm{dgm}\left(\mathrm{Filt}\left(X_{n}\right)\right)\right)\right]\leq C{\left(\frac{\log n}{n}\right)}^{1/b}$$

where the constant C only depends on a and b.

Under additional technical assumptions, the corresponding lower bound can be shown (up to a logarithmic term) (see Chazal et al. (2014)). By applying stability results, similar consistency results can be easily derived under alternative generative models as soon as a consistent estimator of the support under the Hausdorff metric is known. For instance, from the results of the study by Genovese et al. (2012) about Hausdorff support estimation under additive noise, it can be deduced that the minimax convergence rates for the persistence diagram estimation are faster than (log  n)^−1/2^. Moreover, as soon as a stability result is available for some given representation of persistence, similar consistency results can be directly derived from the consistency for persistence diagrams.

#### Estimation of the Persistent Homology of Functions

Theorem 7 opens the door to the estimation of the persistent homology of functions defined on $R^{d}$ , on a submanifold of $R^{d}$ or, more generally, on a metric space. The persistent homology of regression functions has also been studied by Bubenik et al. (2010). The alternative approach of Bobrowski et al. (2014), which was based on the inclusion map between nested pairs of estimated level sets, can be applied with kernel density and regression kernel estimators to estimate persistence homology of density functions and regression functions. Another direction of research on this topic concerns various versions of robust TDA. One solution is to study the persistent homology of the upper-level sets of density estimators (Fasy et al., 2014b). A different approach, more closely related to the distance function, but robust to noise, consists in studying the persistent homology of the sublevel sets of the distance to measure defined in Section 4.4 (Chazal et al., 2017).

### 6.2 Statistic of Persistent Homology Computed on a Point Cloud

**FIGURE 13:**
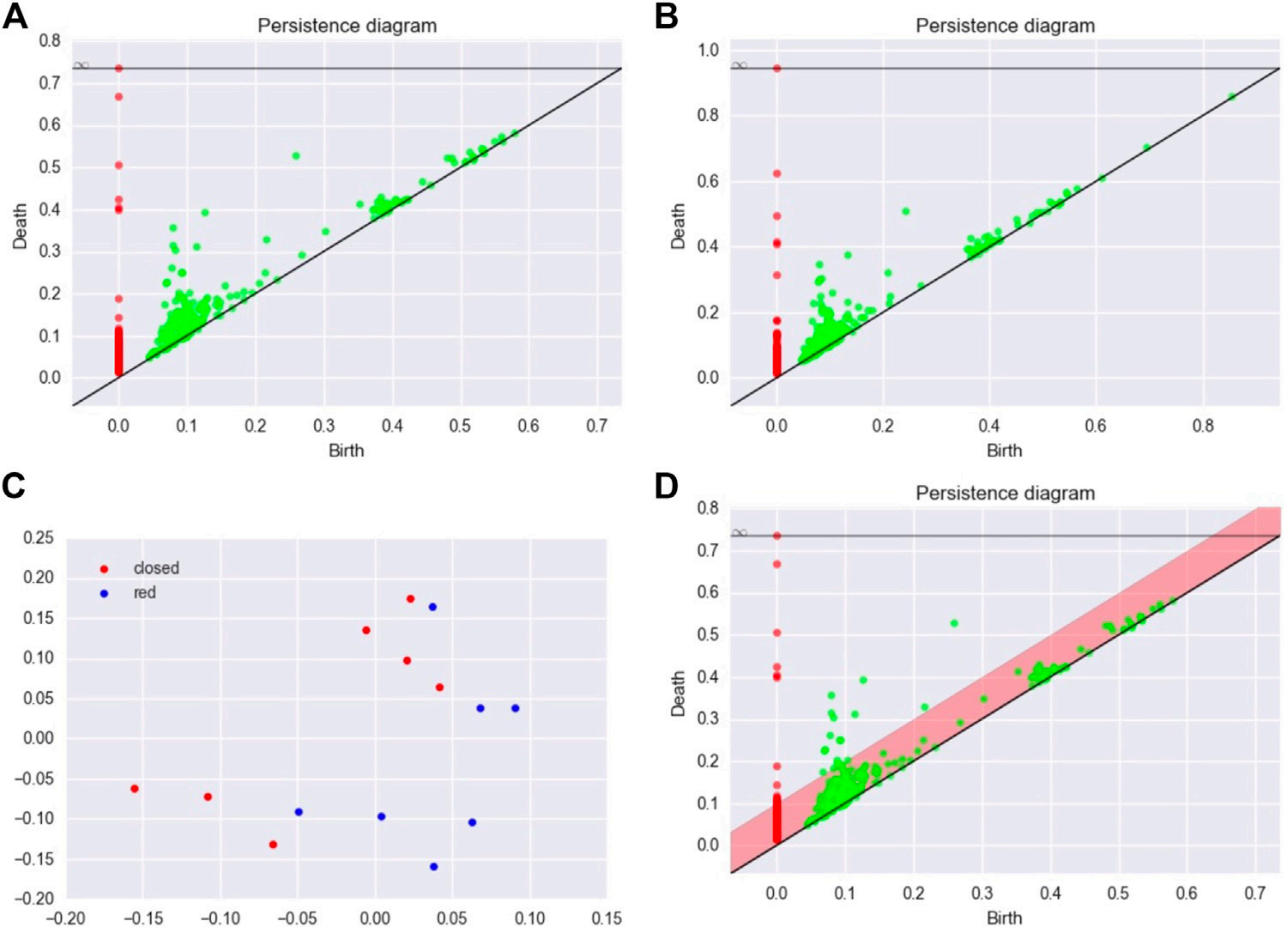
**(A,B)** Two persistence diagrams for two configurations of MBP. **(C)** MDS configuration for the matrix of bottleneck distances. **(D)** Persistence diagram and confidence region for the persistence diagram of an MBP.

For many applications, in particular when the support of the point cloud is not drawn on or close to a geometric shape, persistence diagrams can be quite complex to analyze. In particular, many topological features are closed to the diagonal. Since they correspond to topological structures that die very soon after they appear in the filtration, these points are generally considered as noise (see Figure 13 for an illustration). Confidence regions of persistence diagrams are rigorous answers to the problem of distinguishing between the signal and the noise in these representations.

The stability results given in Section 5.7 motivate the use of the bottleneck distance to define confidence regions. However, alternative distances in the spirit of Wasserstein distances can be proposed too. When estimating a persistence diagram dgm with an estimator $\hat{\mathrm{dgm}}$ , we typically look for some value η_α_ such that

$$P\left(d_{b}\left(\hat{\mathrm{dgm}},\mathrm{dgm}\right)\geq \eta_{\alpha }\right)\leq \alpha ,$$

for α ∈ (0, 1). Let B_α_ be the closed ball of radius α for the bottleneck distance, centered at $\hat{\mathrm{dgm}}$ in the space of persistence diagrams. Following Fasy et al. (2014b), we can visualize the signatures of the points belonging to this ball in various ways. One first option is to center a box of a side length of 2α at each point of the persistence diagram $\hat{\mathrm{dgm}}$ . An alternative solution is to visualize the confidence set by adding a band at (vertical) distance η_α_/2 from the diagonal (the bottleneck distance being defined for the ℓ_∞_ norm) (see Figure 13 for an illustration). The points outside the band are then considered as significant topological features (see Fasy et al. (2014b) for more details).

Several methods have been proposed in the study by Fasy et al. (2014b) to estimate η_α_ in different frameworks. These methods mainly rely on stability results for persistence diagrams; confidence sets for diagrams can be derived from confidence sets in the sample space.

#### Subsampling Approach

This method is based on a confidence region for the support K of the distribution of the sample in the Hausdorff distance. Let ${\tilde{X}}_{b}$ be a subsample of size b drawn from the sample ${\tilde{X}}_{n}$ , where b = o(n/logn). Let q_b_(1 − α) be the quantile of the distribution of $\mathrm{Haus}\left({\tilde{X}}_{b},X_{n}\right)$ . Take ${\hat{\eta }}_{\alpha }≔2{\hat{q}}_{b}\left(1-\alpha \right)$ , where ${\hat{q}}_{b}$ is an estimation q_b_(1 − α) using a standard Monte Carlo procedure. Under a (a, b) standard assumption and for an n large enough, Fasy et al. (2014b) showed that

$$\begin{array}{ccc} P\left(d_{b}\left(\mathrm{dgm}\left(\mathrm{Filt}\left(K\right)\right),\mathrm{dgm}\left(\mathrm{Filt}\left(X_{n}\right)\right)\right)>{\hat{\eta }}_{\alpha }\right) & \leq & P\mathrm{Haus}\left(K,X_{n}\right)>{\hat{\eta }}_{\alpha }\\ & \leq & \alpha +O{\left(\frac{b}{n}\right)}^{1/4}. \end{array}$$

#### Bottleneck Bootstrap

The stability results often lead to conservative confidence sets. An alternative strategy is the bottleneck bootstrap introduced in the study by Chazal et al. (2016b). We consider the general setting where a persistence diagram $\hat{\mathrm{dgm}}$ is defined from the observation (X_1_, … , X_n_) in a metric space. This persistence diagram corresponds to the estimation of an underlying persistence diagram dgm, which can be related, for instance, to the support of the measure, or to the sublevel sets of a function related to this distribution (for instance, a density function when the X_i_’s are in $R^{d}$ ). Let $\left(X_{1}^{*},\ldots ,X_{n}^{*}\right)$ be a sample from the empirical measure defined from the observations (X_1_, … , X_n_). Let also ${\hat{\mathrm{dgm}}}^{*}$ be the persistence diagram derived from this sample. We can then take for η_α_ the quantity ${\hat{\eta }}_{\alpha }$ defined by

$$P\left(d_{b}\left({\hat{\mathrm{dgm}}}^{*},\hat{\mathrm{dgm}}\right)>{\hat{\eta }}_{\alpha }\;|\;X_{1},\ldots ,X_{n}\right)=\alpha .$$ (7)

Note that ${\hat{\eta }}_{\alpha }$ can be easily estimated using Monte Carlo procedures. It has been shown in the study by Chazal et al. (2016b) that the bottleneck bootstrap is valid when computing the sublevel sets of a density estimator.

#### Bootstrapping Persistent Betti Numbers

As already mentioned, confidence regions based on stability properties of persistence may lead to very conservative confidence regions. Based on the concepts of stabilizing statistics Penrose and Yukich (2001), asymptotic normality for persistent Betti numbers has been shown recently by Krebs and Polonik (2019) and Roycraft et al. (2020) under very mild conditions on the filtration and the distribution of the sample cloud. In addition, bootstrap procedures are also shown to be valid in this framework. More precisely, a smoothed bootstrap procedure together with a convenient rescaling of the point cloud seems to be a promising approach for boostrapping TDA features from point cloud data.

### 6.3 Statistic for a Family of Persistent Diagrams or Other Representations

Up to now in this section, we were only considering statistics based on one single observed persistence diagram. We now consider a new framework where several persistence diagrams (or other representations) are available, and we are interested in providing the central tendency, confidence regions, and hypothesis tests for topological descriptors built on this family.

#### 6.3.1 Central Tendency for Persistent Homology

##### Mean and Expectations of Distributions of Diagrams

The space of persistence diagrams being a general metric space but not a Hilbert space, the definition of a mean persistence diagram is not obvious and unique. One first natural approach to defining a central tendency in this context is to consider Fréchet means of distributions of diagrams. Their existence has been proven in the study by Mileyko et al. (2011), and they have also been characterized in the study by Turner et al. (2014a). However, they may not be unique, and they turn out to be difficult to compute in practice. To partly overcome these problems, different approaches have been recently proposed based on numerical optimal transport Lacombe et al. (2018) or linear representations and kernel-based methods Divol and Chazal (2020).

##### Topological Signatures From Subsamples

Central tendency properties of persistent homology can also be used to compute topological signatures for very large data sets, as an alternative approach to overcome the prohibitive cost of persistence computations. Given a large point cloud, the idea is to extract many subsamples, to compute the persistence landscape for each subsample, and then to combine the information.

For any positive integer m, let X = {x_1_, … , x_m_} be a sample of m points drawn from a measure μ in a metric space M and which support is denoted by $X_{\mu }$ . We assume that the diameter of $X_{\mu }$ is finite and upper bounded by $\frac{T}{2}$ , where T is the same constant as in the definition of persistence landscapes in Section 5.4. For ease of exposition, we focus on the case k = 1 and the set λ(t) = λ(1, t). However, the results we present in this section hold for k > 1. The corresponding persistence landscape (associated with the persistence diagram of the Čech or Rips–Vietoris filtration) is λ_X_ and we denote by $\Psi_{\mu }^{m}$ the measure induced by μ^⊗m^ on the space of persistence landscapes. Note that the persistence landscape λ_X_ can be seen as a single draw from the measure $\Psi_{\mu }^{m}$ . The point-wise expectations of the (random) persistence landscape under this measure is defined by $E_{\Psi_{\mu }^{m}}\left[\lambda_{X}\left(t\right)\right],t\in \left[0,T\right]$ . The average landscape $E_{\Psi_{\mu }^{m}}\left[\lambda_{X}\right]$ has a natural empirical counterpart, which can be used as its unbiased estimator. Let $S_{1}^{m},\ldots ,S_{\ell }^{m}$ be ℓ independent samples of size m from μ^⊗m^. We define the empirical average landscape as

$$\bar{\lambda_{\ell }^{m}}\left(t\right)=\frac{1}{b}\overset{b}{\underset{i=1}{\sum }}\lambda_{S_{i}^{m}}\left(t\right),\;\text{ for all }t\in \left[0,T\right],$$ (8)

and propose to use $\bar{\lambda_{\ell }^{m}}$ to estimate $\lambda_{X_{\mu }}$ . Note that computing the persistent homology of $X_{n}$ is O(exp(n)), whereas computing the average landscape is O(b exp(m)).

Another motivation for this subsampling approach is that it can also be applied when μ is a discrete measure with the support $X_{N}=\left\{x_{1},\ldots ,x_{N}\right\}$ lying in a metric space M. This framework can be very common in practice, when a continuous (but unknown) measure is approximated by a discrete uniform measure μ_N_ on $X_{N}$ .

The average landscape $E_{\Psi_{\mu }^{m}}\left[\lambda_{X}\right]$ is an interesting quantity on its own, since it carries some stable topological information about the underlying measure μ, from which the data are generated.

**Theorem 11.** *[* *Chazal et al. (2015a)* *] Let X ∼ μ* ^*⊗m*^ *and Y ∼ ν* ^*⊗m*^ *, where μ and ν are two probability measures on M. For any p ≥ 1, we have*

$${\left\|E_{\Psi_{\mu }^{m}}\left[\lambda_{X}\right]-E_{\Psi_{\nu }^{m}}\left[\lambda_{Y}\right]\right\|}_{\infty }\leq 2\;m^{\frac{1}{p}}W_{p}\left(\mu ,\nu \right),$$

where W_p_ is the pth Wasserstein distance on M.

The result of Theorem 11 is useful for two reasons. First, it tells us that for a fixed m, the expected “topological behavior” of a set of m points carries some stable information about the underlying measure from which the data are generated. Second, it provides a lower bound for the Wasserstein distance between two measures, based on the topological signature of samples of m points.

#### 6.3.2 Asymptotic Normality

As in the previous section, we consider several persistence diagrams (or other representations). The next step after giving central tendency descriptors of persistence homology is to provide asymptotic normality results for these quantities together with bootstrap procedures to derive confidence regions. It is of course easier to show such results for functional representations of persistence. In the studies by Chazal et al. (2015b), Chazal et al. (2015c), following this strategy, confidence bands for landscapes are proposed from the observation of landscapes λ_1_, … , λ_N_ drawn i. i. d. from a random distribution in the space of landscapes. The asymptotic validity and the uniform convergence of the multiplier bootstrap is shown in this framework. Note that similar results can also be proposed for many representations of persistence, in particular by showing that the corresponding functional spaces are Donsker spaces.

### 6.4 Other Statistical Approaches to Topological Data Analysis

Statistical approaches for tda are seeing an increasing interest and many others have been proposed in recent years or are still subject to active research activities, as illustrated in the following non-exhaustive list of examples.

#### Hypothesis Testing

Several methods have been proposed for hypothesis testing procedures for persistent homology, mostly based on permutation strategies and for two-sample testing. Robinson and Turner (2017) focused on pairwise distances of persistence diagrams, whereas Berry et al. (2020) studied more general functional summaries. Hypothesis tests based on kernel approaches have been proposed in the study by Kusano (2019). A two-stage hypothesis test of filtering and testing for persistent images was also presented in the study by Moon and Lazar (2020).

#### Persistence Homology Transform

The representations introduced before are all transformations derived from the persistence diagram computed from a fixed filtration built over a data set. The persistence homology transform introduced in the studies by Curry et al. (2018), Turner et al. (2014b) to study shapes in $R^{d}$ takes a different path by looking at the persistence homology of the sublevel set filtration induced by the projection of the considered shape in each direction in $R^{d}$ . It comes with several interesting properties; in particular, the persistence homology transform is a sufficient statistic for distributions defined on the set of geometric and finite simplicial complexes embedded in $R^{d}$ .

#### Bayesian Statistics for Topological Data Analysis

A Bayesian approach to persistence diagram inference has been proposed in the study by Maroulas et al. (2020) by viewing a persistence diagram as a sample from a point process. This Bayesian method computes the point process posterior intensity based on a Gaussian mixture intensity for the prior.

### 6.5 Persistent Homology and Machine Learning

Using tda and, more specifically, persistent homology for machine learning is a subject that attracts a lot of information and generated an intense research activity. Although the recent progress in this area goes far beyond the scope of this article, we briefly introduce the main research directions with a few references to help the newcomer to the field to get started.

#### Topological Data Analysis for Exploratory Data Analysis and Descriptive Statistics

In some domains, tda can be fruitfully used as a tool for exploratory analysis and visualization. For example, the Mapper algorithm provides a powerful approach to exploring and visualizing the global topological structure of complex data sets. In some cases, persistence diagrams obtained from data can be directly interpreted and exploited for better understanding of the phenomena from which the data have been generated. This is, for example, the case in the study of force fields in granular media (Kramar et al., 2013) or of atomic structures in glass (Nakamura et al., 2015) in material science, in the study of the evolution of convection patterns in fluid dynamics (Kramár et al., 2016), and in machining monitoring (Khasawneh and Munch, 2016) or in the analysis of nanoporous structures in chemistry (Lee et al., 2017) where topological features can be rather clearly related to specific geometric structures and patterns in the considered data.

#### Persistent Homology for Feature Engineering

There are many other cases where persistence features cannot be easily or directly interpreted but present valuable information for further processing. However, the highly nonlinear nature of diagrams prevents them from being immediately used as standard features in machine learning algorithms.

Persistence landscapes and linear representations of persistence diagrams offer a first option to convert persistence diagrams into elements of a vector space that can be directly used as features in classical machine learning pipelines. This approach has been used, for example, for protein binding (Kovacev-Nikolic et al., 2016), object recognition (Li et al., 2014), or time series analysis. In the same vein, the construction of kernels for persistence diagrams that preserve their stability properties has recently attracted some attention. Most of them have been obtained by considering diagrams as discrete measures in $R^{2}$ . Convolving a symmetrized (with respect to the diagonal) version of persistence diagrams with a 2D Gaussian distribution, Reininghaus et al. (2015) introduced a multiscale kernel and applied it to shape classification and texture recognition problems. Considering the Wasserstein distance between projections of persistence diagrams on lines, Carriere et al. (2017) built another kernel and tested its performance on several benchmarks. Other kernels, still obtained by considering persistence diagrams as measures, have also been proposed in the study by Kusano et al. (2017).

Various other vector summaries of persistence diagrams have been proposed and then used as features for different problems. For example, basic summaries were considered in the study by Bonis et al. (2016) and combined with quantization and pooling methods to address nonrigid shape analysis problems; Betti curves extracted from persistence diagrams were used with one-dimensional convolutional neural networks (CNNs) to analyze time-dependent data and recognize human activities from inertial sensors in the studies by Dindin et al. (2020), Umeda (2017); persistence images were introduced in the study by Adams et al. (2017) and were considered to address some inverse problems using linear machine learning models in the study by Obayashi et al. (2018).

The kernels and vector summaries of persistence diagrams mentioned above are built independently of the considered data analysis or learning task. Moreover, it appears that in many cases, the relevant topological information is not carried by the whole persistence diagram but is concentrated in some localized regions that may not be obvious to identify. This usually makes the choice of a relevant kernel or vector summary very difficult for the user. To overcome this issue, various authors have proposed learning approaches that allow us to learn the relevant topological features for a given task. In this direction, Hofer et al. (2017) proposed a deep learning approach to learn the parameters of persistence image representations of persistence diagrams, while Kim et al. (2020) introduced a neural network layer for persistence landscapes. In the study by Carrière et al. (2020a), the authors introduced a general neural network layer for persistence diagrams that can be either used to learn an appropriate vectorization or directly integrated in a deep neural network architecture. Other methods, inspired from *k*-means, propose unsupervised methods to vectorize persistence diagrams (Royer et al., 2021; Zieliński et al., 2010), some of them coming with theoretical guarantees (Chazal et al., 2020).

#### Persistent Homology for Machine Learning Architecture Optimization and Model Selection

More recently, tda has seen new developments in machine learning where persistent homology is no longer used for feature engineering but as a tool to design, improve, or select models (see Carlsson and Gabrielsson (2020), Chen et al. (2019), Gabrielsson and Carlsson (2019), Hofer et al. (2019a), Moor et al. (2020), Ramamurthy et al. (2019), Rieck et al. (2019)). Many of these tools rely on the introduction of loss or regularization functions depending on persistent homology features, raising the problem of their optimization. Building on the powerful tools provided by software libraries such as PyTorch or TensorFlow, practical methods allowing us to encode and optimize a large family of persistence-based functions have been proposed and experimented on (Poulenard et al., 2018; Gabrielsson et al., 2020). A general framework for persistence-based function optimization based on stochastic subgradient descent algorithms with convergence guarantees has been recently proposed and implemented in an easy-to-use software tool (Carriere et al., 2020b). With a different perspective, another theoretical framework to study the differentiable structure of functions of persistence diagrams has been proposed in the study by Leygonie et al. (2021).

## 7 Topological Data Analysis for Data Sciences With the GUDHI Library

In this section, we illustrate TDA methods using the Python library GUDHI^11^ (Maria et al., 2014) together with popular libraries such as NumPy (Walt et al., 2011), scikit-learn (Pedregosa et al., 2011), and pandas (McKinney, 2010). This section aims at demonstrating that the topological signatures of TDA can be easily computed and exploited using GUDHI. More illustrations with Python notebooks can be found in the tutorial GitHub^12^ of GUDHI.

### 7.1 Bootstrap and Comparison of Protein Binding Configurations

This example is borrowed from Kovacev-Nikolic et al. (2016). In this article, persistent homology is used to analyze protein binding, and more precisely, it compares closed and open forms of the maltose-binding protein (MBP), a large biomolecule consisting of 370 amino acid residues. The analysis is not based on geometric distances in $R^{3}$ but on a metric of dynamical distances defined by

$D_{ij}=1-|C_{ij}|,$ where C is the correlation matrices between residues. The data can be downloaded at this link^13^.

import numpy as np

import gudhi as gd

import pandas as pd

import seaborn as sns

corr_protein = pd.read_csv(“mypath/1anf.corr_1.txt”, header=None, delim_whitespace=True)

dist_protein_1 = 1− np.abs(corr_protein_1.values)

rips_complex_1 = gd.RipsComplex(distance_matrix=dist_protein_1, max_edge_length=1.1)

simplex_tree_1 = rips_complex_1.create_simplex_tree(max_dimension=2)

diag_1 = simplex_tree_1.persistence()

gd.plot_persistence_diagram(diag_1)

For comparing persistence diagrams, we use the bottleneck distance. The block of statements given below computes persistence intervals and computes the bottleneck distance for zero-homology and one-homology as follows:

interv0_1 = simplex_tree_1.persistence_intervals_in_dimension(0)

interv0_2 = simplex_tree_2.persistence_intervals_in_dimension(0)

bot0 = gd.bottleneck_distance(interv0_1,interv0_2)

interv1_1 = simplex_tree_1.persistence_intervals_in_dimension(1)

interv1_2 = simplex_tree_2.persistence_intervals_in_dimension(1)

bot1 = gd.bottleneck_distance(interv1_1,interv1_2)

In this way, we can compute the matrix of bottleneck distances between the fourteen MBPs. Finally, we apply a multidimensional scaling method to find a configuration in $R^{2}$ which almost matches with the bottleneck distances (see Figure 13C). We use the scikit-learn library for the MDS as follows:

import matplotlib.pyplot as plt

from sklearn import manifold

mds = manifold.MDS(n_components=2, dissimilarity=“precomputed”)

config = mds.fit(M).embedding_

plt.scatter(config [0:7,0], config [0:7, 1], color=‘red’, label=“closed”)

plt.scatter(config [7:l,0], config [7:l, 1], color=‘blue’, label=“red”)

plt.legend(loc=1)

We now define a confidence band for a diagram using the bottleneck bootstrap approach. We resample over the lines (and columns) of the matrix of distances, and we compute the bottleneck distance between the original persistence diagram and the bootstrapped persistence diagram. We repeat the procedure many times, and finally, we estimate the quantile 95% of this collection of bottleneck distances. We take the value of the quantile to define a confidence band on the original diagram (see Figure 13D). However, such a procedure should be considered with caution because as far as we know, the validity of the bottleneck bootstrap has not been proven in this framework.

### 7.2 Classification for Sensor Data

In this experiment, the 3D acceleration of 3 walkers (A, B, and C) has been recorded using the sensor of a smartphone^14^. Persistence homology is not sensitive to the choice of axes, and so no preprocessing is necessary to align the 3 time series according to the same axis. From these three time series, we have picked, at random, sequences of 8 s in the complete time series, that is, 200 consecutive points of acceleration in $R^{3}$ . For each walker, we extract 100 time series in this way. The next block of statements computes the persistence for the alpha complex filtration for data_A_sample, one of the 100 time series of acceleration of Walker A.

alpha_complex_sample = gd.AlphaComplex(points = data_A_sample)

simplex_tree_sample = alpha_complex_sample.create_simplex_tree(max_alpha_square=0.3)

diag_Alpha = simplex_tree_sample.persistence()

**FIGURE 14:**
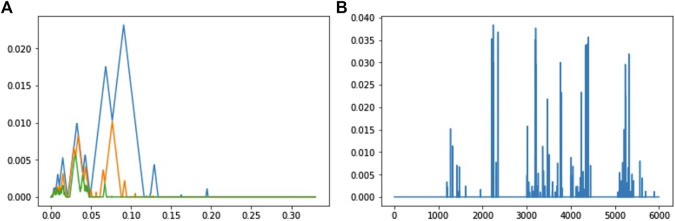
**(A)** First three landscapes for zero-homology of the alpha shape filtration defined for a time series of acceleration of Walker A. **(B)** Variable importances of the landscape coefficients for the classification of walkers. The first 3,000 coefficients correspond to the three landscapes of dimension 0 and the last 3,000 coefficients to the three landscapes of dimension 1. There are 1,000 coefficients per landscape. Note that the first landscape of dimension 0 is always the same using the Rips complex (a trivial landscape), and consequently, the corresponding coefficients have a zero-importance value.

From diag_Alpha, we can then easily compute and plot the persistence landscapes (see Figure 14A). For all 300 time series, we compute the persistence landscapes for dimensions 0 and 1, and we compute the first three landscapes for the 2 dimensions. Moreover, each persistence landscape is discretized on 1,000 points. Each time series is thus described by 6,000 topological variables. To predict the walker from these features, we use a random forest (Breiman, 2001), which is known to be efficient in such a high-dimensional setting. We split the data into train and test samples at random several times. We finally obtain an averaged classification error of around 0.95. We can also visualize the most important variables in the random forest (see Figure 14B).

## 8 Discussion

In this introductory article, we propose an overview of the most standard methods in the field of topological data analysis. We also provide a presentation of the mathematical foundations of TDA, on the topological, algebraic, geometric, and statistical aspects. The robustness of TDA methods (coordinate invariance and deformation invariance) and the compressed representation of data they offer make their use very interesting for data analysis, machine learning, and explainable AI. Many applications have been proposed in this direction during the last few years. Finally, TDA constitutes an additional possible approach in the data scientist toolbox.

Of course, TDA is suited to address all kinds of problems. Practitioners may face several potential issues when applying TDA methods. On the algorithmic aspects, computing persistence homology can be time and resource consuming. Even if there is still room for improvement, recent computational advances have enabled TDA to be an effective method for data science, thanks to libraries like GUDHI, for example. Moreover, combing TDA using quantization methods, graph simplification, or dimension reduction methods may reduce the computational cost of the TDA algorithms. Another potential problem we can face with TDA is that returning to the data point to interpret the topological signatures can be tricky because these signatures correspond to classes of equivalence of cycles. This can be a problem when there is a need to identify which part of the point cloud “has created” a given topological signature. TDA is in fact more suited to solving data science problems dealing with a family of point clouds, each data point being described by its persistent homology. Finally, the topological and geometric information that can be extracted from the data is not always efficient for solving a given problem in the data sciences alone. Combining topological signatures with other types of descriptors is generally a relevant approach.

Today, TDA is an active field of research, at the crossroads of many scientific fields. In particular, there is currently an intense effort to effectively combine machine learning, statistics, and TDA. In this perspective, we believe that there is still a need for statistical results which demonstrate and quantify the interest of these data science approaches based on TDA.

## Footnotes

1
https://gudhi.inria.fr/

2
http://www.mrzv.org/software/dionysus/

3
https://bitbucket.org/phat-code/phat

4
https://github.com/DIPHA/dipha

5
https://giotto-ai.github.io/gtda-docs/0.4.0/library.html

6 As an example, if M is a smooth compact submanifold, then reach_0_(ϕ) is always positive and known as the reach of M Federer (1959).

7 Recall that the symmetric difference of two sets A and B is the set AΔB = (A \ B) ∪ (B \ A).

8 Notice that as we are considering coefficients in $Z_{2}$ , here −1 = 1 and thus (−1)^i^ = 1 for any i.

9 See Villani (2003) for a definition of the Wasserstein distance

10 We take here the convention that for r < 0, ${\mathrm{Rips}}_{r}\left(X\right)={\mathrm{Cech}}_{r}\left(X\right)=\emptyset$

11
http://gudhi.gforge.inria.fr/python/latest/

12
https://github.com/GUDHI/TDA-tutorial

13
https://www.researchgate.net/publication/301543862_corr

14 The dataset can be downloaded at the link http://bertrand.michel.perso.math.cnrs.fr/Enseignements/TDA/data_acc
